# Recent structural evolution of lactam- and imide-functionalized polymers applied in organic field-effect transistors and organic solar cells

**DOI:** 10.1039/d1sc01711j

**Published:** 2021-05-07

**Authors:** Yankai Zhou, Weifeng Zhang, Gui Yu

**Affiliations:** Beijing National Laboratory for Molecular Sciences, CAS Research/Education Centre for Excellence in Molecular Sciences, Institute of Chemistry, Chinese Academy of Sciences Beijing 100190 P. R. China yugui@iccas.ac.cn zhangwf@iccas.ac.cn; School of Chemical Sciences, University of Chinese Academy of Sciences Beijing 100049 P. R. China

## Abstract

Organic semiconductor materials, especially donor–acceptor (D–A) polymers, have been increasingly applied in organic optoelectronic devices, such as organic field-effect transistors (OFETs) and organic solar cells (OSCs). Plenty of high-performance OFETs and OSCs have been achieved based on varieties of structurally modified D–A polymers. As the basic building block of D–A polymers, acceptor moieties have drawn much attention. Among the numerous types, lactam- and imide-functionalized electron-deficient building blocks have been widely investigated. In this review, the structural evolution of lactam- or imide-containing acceptors (for instance, diketopyrrolopyrrole, isoindigo, naphthalene diimide, and perylene diimide) is covered and their representative polymers applied in OFETs and OSCs are also discussed, with a focus on the effect of varied structurally modified acceptor moieties on the physicochemical and photoelectrical properties of polymers. Additionally, this review discusses the current issues that need to be settled down and the further development of new types of acceptors. It is hoped that this review could help design new electron-deficient building blocks, find a more valid method to modify already reported acceptor units, and achieve high-performance semiconductor materials eventually.

## Abstract 2

This review highlights the recent structural evolution of lactam- and imide-functionalized polymers applied in organic field-effect transistors and organic solar cells.

## Graphical abstract

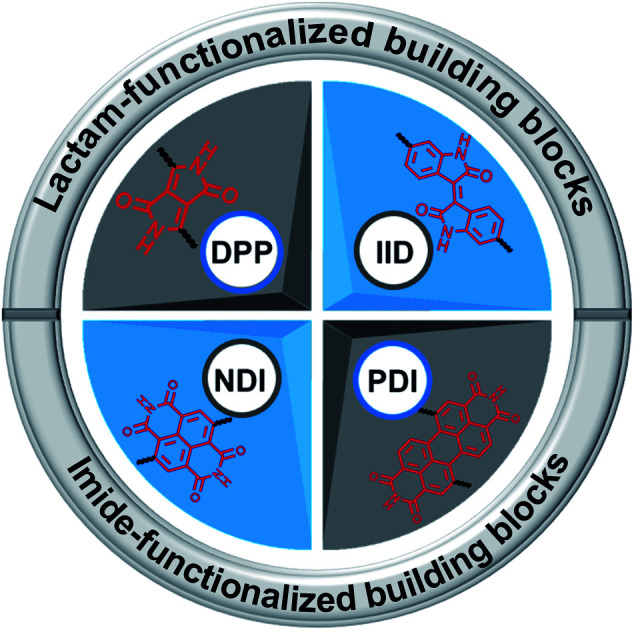

## 1. Introduction

Since chlorine-doped aromatic carbohydrate films were firstly found to be conductive in the 1950s,^1,2^ organic semiconducting materials have attracted a large amount of interest and attention from researchers around the world. In 1986, the first organic field-effect transistor (OFET) based on polythiophene was fabricated, from then on a large number of organic semiconducting materials, possessing outstanding properties and novel chemical structures, have been designed and synthesized.^3^ In recent decades, the strategy of combining electron-rich (donor) and electron-deficient (acceptor) units alternately together has become increasingly popular and has been applied to obtain all kinds of D–A type polymeric semiconductors. As is well known, the structure of the acceptor unit has a great influence on its corresponding polymer, no matter physicochemical properties or optoelectronic performances. Therefore, the design of novel acceptors and their structural optimization have received considerable attention. In recent years, many structurally varied acceptors were synthesized and numerous high-performance semiconducting polymers were reported. Among the well-known electron-deficient units and the corresponding polymers lactam- and imide-based acceptors and corresponding polymers have been attracting the attention of scientists until now, due to their excellent optoelectronic properties and wide applications in high-performance OFETs and organic solar cells (OSCs).

Lactam is a cyclic amide, which consists of a carbonyl group adjacent to an amino group. Imide has the same structure with one more carbonyl group connected to the amino group. These two groups were increasingly used to construct acceptors for high-performance semiconducting polymers, which can be explained by the following points: firstly, the nitrogen atom in the structure adopts sp^2^ hybridization, with its p-orbital conjugation with the π-orbital from the adjacent carbonyl group. The formed p–π conjugation facilitates evenly distributed electron density and better planarity, both of which could positively impact the polymer properties.^4–7^ Secondly, constructing acceptor units with strong electron-withdrawing lactam or imide groups provides the molecules and polymers with low-lying energy levels of the lowest unoccupied molecular orbital (LUMO), thus promoting electron injection efficiency and enhancing the device air stability.^8–10^ Thirdly, a variety of side chains can be substituted on the amide nitrogen atom to adjust the polymer solubility, film morphology, and the self-assembly of the side chain.^11–13^ Some functional side chains were also incorporated into polymers to fabricate devices with a specific function.^14–16^ In recent years, numerous lactam- and imide-functionalized polymers with high carrier mobilities have been reported. Considering the mobility of silicon FETs, which is around 1 cm^2^ V^−1^ s^−1^, the ultra-high hole mobility of over 14.0 cm^2^ V^−1^ s^−1^ and the balanced hole/electron mobilities around 6.5 cm^2^ V^−1^ s^−1^ achieved by polymer semiconductors have made considerable progress. Despite that some of these preferable performances were probably extracted from the saturation region, they are still valid tools to assess the application potential of different polymers. Besides, the PCE values of OSCs were improved to 14%, even higher. All the above impressive advances are made by those lactam or imide-containing polymers.

In this progress review, we first summarize the recent structural evolution of classic lactam-containing electron-deficient building blocks, mainly focus on diketopyrrolopyrrole (DPP) and isoindigo (IID) units, and investigate how such modification methods eventually lead to the different physicochemical properties and optoelectronic performances of their corresponding polymers. Then, various imide-functionalized acceptors (naphthalene diimide (NDI), perylene diimide (PDI), *etc.*) and polymers with modified molecular structures are also covered. Finally, we discuss the development strategy and feasible optimization methods for current acceptors and polymers, followed by the current issues we are meeting and the future development of analogous molecules.

## 2. Polymers with lactam moieties

As the commonly used structural building block, the lactam moiety is famous for its strong electron-withdrawing nature and changeable substituted sidechains. Owing to their outstanding features, lactam-functionalized acceptor building blocks have been widely used in obtaining high-performance small molecules and polymer semiconductors. Among the numerous building blocks, diketopyrrolopyrrole (DPP) and isoindigo (IID) units are the most representative lactam-containing acceptor units. It is convenient to regulate their molecular solubility and electron deficiency because of their adjustable molecular frameworks and less modification complexity.^17–20^ Therefore, those two building blocks have developed rapidly in recent years. A large number of derivatives and analogs of DPP and IID units are designed and numerous high-performance DPP- and IID-based polymers have been reported in recent two decades, which we introduce below.

### 2.1. DPP-based polymers

As a popular molecule in pigment chemistry, DPP contains two lactam groups and has been increasingly applied in the field of organic electronics during the past several years.^21–23^ Multiple polymeric materials have been designed and synthesized based on the DPP unit, due to its proper frontier molecular orbital (FMO) energy levels and its adjustable molecular structure. To sum up, there are two main ways to construct a new type of electron-accepting DPP-based unit. One is the introduction of flanked aromatic rings on both sides of the DPP core according to the methodology proposed by Rochat and co-workers,^24^ such as thiophene, furan, benzene, and other aromatic units, for improved molecular planarity and electron deficiency. The other strategy is adjusting the molecular structure of the DPP core, no matter to increase the conjugation length or to change the way of *N*-substitution. Such methodologies are beneficial to obtain varieties of high-performance polymers containing DPP or its multiple derivatives. Accordingly, there are mainly three types of structures for DPP derivatives, (i) having symmetrical flanking units; (ii) having asymmetrical flanking units; (iii) having modified DPP core structures.

#### 2.1.1. Polymers with symmetrically flanked DPP units

**Fig. 1:**
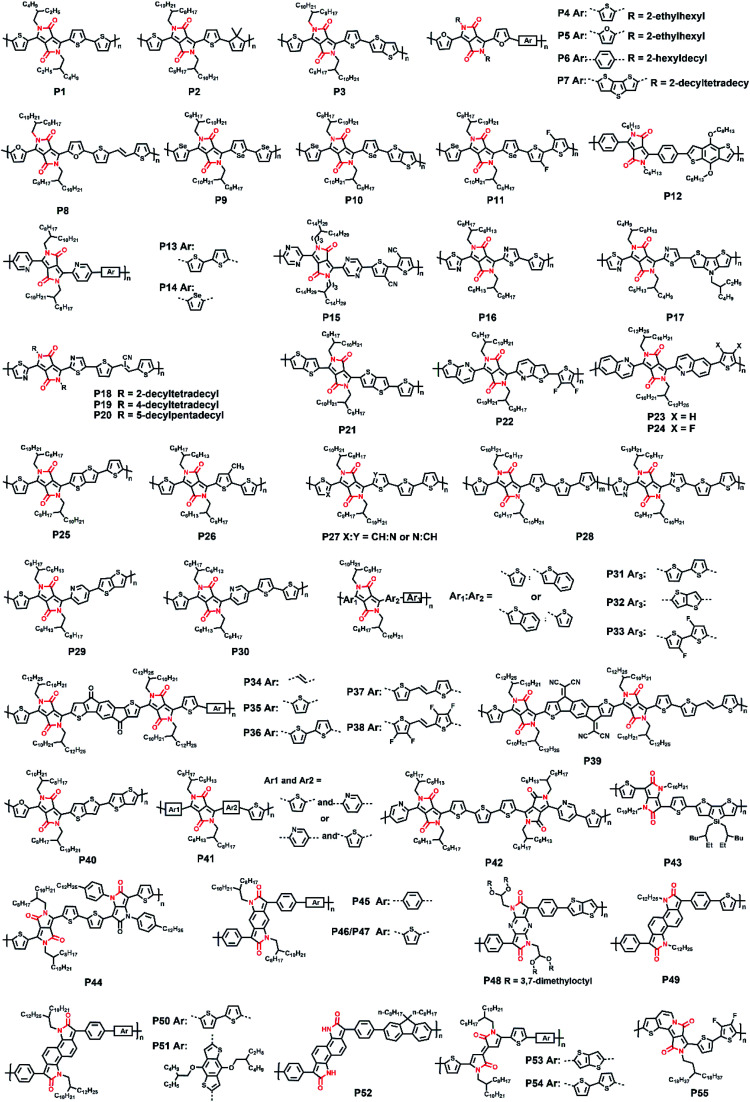
The molecular structures of polymers **P1–P55**.

**Fig. 2:**
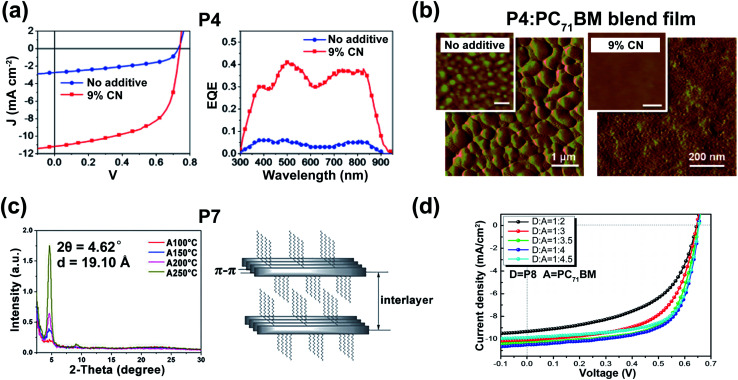
(a) The *J*–*V* curves (left) and the external quantum efficiency spectra (right) of the optimized devices based on **P4**:PC_71_BM blend films (weight ratio = 1 : 3). Reproduced from ref. 29 with permission from the American Chemical Society, copyright 2010. (b) The AFM phase images of **P4**:PC_71_BM blend films (weight ratio = 1 : 3). Reproduced from ref. 29 with permission from the American Chemical Society, copyright 2010. (c) XRD data (left) and the illustration of lamellar structure (right) of **P7** film. Reproduced from ref. 31 with permission from the Royal Society of Chemistry, copyright 2015. (d) The OSC performances of **P8**:PC_71_BM with different ratios. Reproduced from ref. 32 with permission from the Royal Society of Chemistry, copyright 2017.

**Fig. 3:**
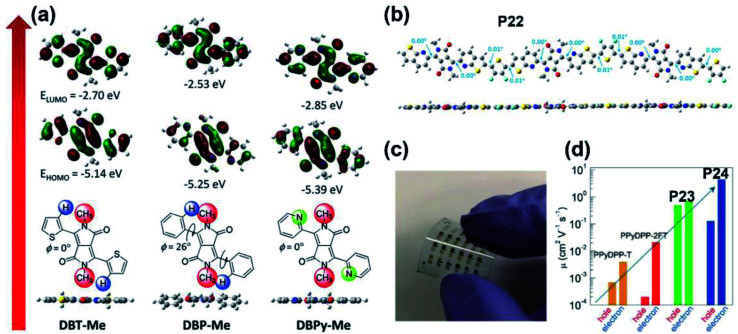
(a) The optimized geometries and FMO energy levels of the thiophene-, phenyl-, and pyridine-flanked DPP monomers with methyl substitution. Reproduced from ref. 37 with permission from Wiley-VCH, copyright 2014. (b) The DFT-simulated backbone geometry of the **P22** trimer. Reproduced from ref. 44 with permission from the American Chemical Society, copyright 2017. (c) The flexible OFET array fabricated on the PET substrate. Reproduced from ref. 45 with permission from Wiley-VCH, copyright 2018. (d) The comparison of ambipolar mobilities between **P23**/**P24** and their pyridine-based counter polymers. Reproduced from ref. 45 with permission from Wiley-VCH, copyright 2018.

The DPP unit was firstly synthesized in 1993 by Chan and co-workers; they investigated the optical properties, charge carrier mobilities, and the photorefractive effect of DPP-based polymers.^25^ However, there are no DPP-based polymeric semiconductors applied in the organic electronics field until Bijleveld and co-workers incorporated the thiophene unit into both sides of DPP and synthesized polymer **P1** based on the dithiophene-DPP unit.^26^**P1** showed balanced ambipolar properties with the hole and electron mobilities of 0.04 and 0.01 cm^2^ V^−1^ s^−1^, respectively (Table 1). Surprisingly, the mixture of **P1** and [6,6]-phenyl-C_71_-butyric acid methyl ester ([70]PCBM or PC_71_BM) also afforded a photoelectric conversion efficiency of 4.7%. Since then, various thiophene-flanked DPP-based polymers with novel donor units have been widely used in organic electronic devices. Fei and co-workers utilized thiophene-flanked DPP to synthesize polymer **P2**.^27^ With copper(i) thiocyanate as the interlayer and penta-fluorobenzene thiol modified gold source and drain electrodes, the **P2** film afforded a max hole mobility of 3.1 cm^2^ V^−1^ s^−1^. The carrier mobilities of polymers containing the thiophene-flanked DPP unit have been greatly enhanced in recent several years. Luo and co-workers used thieno[3,2-*b*]thiophene as the acceptor in the backbone of **P3** and successfully achieved a max hole mobility of 2.1 cm^2^ V^−1^ s^−1^.^28^ More impressively, the additional ionic additive, tetramethylammonium iodide (NMe_4_I), in polymer **P3** facilitated the film interchain packing and formed a stable conformation with **P3**. With the neglectable influence in the ultraviolet-visible (UV-vis) absorption, infrared (IR), and other optical spectra of **P3**, the additional NMe_4_I provided the **P3**-NMe_4_I blend film with larger fiber aggregates, a more ordered side chain packing state, denser polymer interchain stacking, and more importantly a much higher hole mobility up to 26.2 cm^2^ V^−1^ s^−1^. Besides, the **P3**-NMe_4_I blend film maintained a high transport performance for a long period, indicating great device stability. Such results also proved that the proper collocation of the additive and polymers with specific chemical structures could exert favorable charge carrier enhancement. The success of the thiophene flanking strategy encouraged the attempt in other heterocycles, such as the furan ring, which has comparable energy levels and aromaticity to the thiophene unit. Woo and co-workers reported two corresponding polymers **P4** and **P5** based on the furan-flanked DPP unit.^29^ The furan bridge permitted the use of shorter sidechain substitution thus potentially facilitating interchain ordering. The **P4**/PC_61_BM and **P5**/PC_61_BM afforded a power conversion efficiency (PCE) of 3.8% and 3.0%, respectively, when the weight ratio of **P4**(**P5**) : PC_61_BM was 1 : 3. After optimization with an additional 9% 1-chloronaphthalene, the PCE of **P4**(**P5**)/PC_71_BM was improved and the max PCE reached 5.0% (Fig. 2a) due to its fiber-like interpenetrating morphologies (Fig. 2b). Moreover, another furan-DPP-based polymer **P6** achieved balanced ambipolar hole and electron mobilities, with the PCE value of the OSC device still reaching 3.7%.^30^ By changing the donor building block to the dithienothiophene unit, polymer **P7** gave an impressive enhancement in the hole mobility of 3.56 cm^2^ V^−1^ s^−1^, due to its good crystallinity and appropriate interlayer distance (Fig. 2c).^31^ Besides, polymer **P8**, containing furan-DPP and (*E*)-1,2-di-2-thienylethylene units, showed improved OSC performance in comparison with that of **P6**.^32^ It was found that the **P8**:PC_71_BM blend film afforded the max photovoltaic performance when the weight ratio was 1 : 4 (Fig. 2d), with a PCE of 4.56%. Besides the furan ring, the selenophene ring was also chosen as the bridge unit of the DPP core. The high polarizability of the selenophene atom is prone to form the Se⋯O intramolecular interaction in the DPP molecule, thus facilitating high molecular planarity. Shahid and co-workers prepared two polymers **P9** and **P10**.^33^ Both the polymers showed narrower optical band gaps than that of thiophene-DPP-based polymers, facilitating the achievement of balanced electron and hole mobilities of 0.1 cm^2^ V^−1^ s^−1^. By changing the donor to a fluorinated bithiophene unit, Liu and co-workers synthesized the selenophene-DPP-based polymer (**P11**) with a lowered HOMO energy level.^34^ The easier hole carrier injection gave a much improved hole mobility of 0.95 cm^2^ V^−1^ s^−1^ compared with the hole mobilities of **P9** and **P10**. Besides, the additional NMe_4_I contributed a higher hole mobility of up to 1.51 cm^2^ V^−1^ s^−1^ under ambient conditions, indicating its further potential in better FET performance. It is well known that most of the asymmetric five-membered bridges, such as furan, thiophene, and selenophene flanked in the above DPP units, are more electron-rich compared with six-membered aromatic rings. Therefore, synthesizing DPP bridged with six-membered aromatic rings could possibly enhance electron deficiency. The benzene ring, in this regard, has the potential to make a difference. As shown in Fig. 1, polymer **P12** was prepared with phenyl-flanked DPP.^35^ In comparison with thiophene-flanked counter polymers, the electron deficiency of phenyl rings provided **P12** with lower HOMO/LUMO energy levels and smaller band gaps, which led to a PCE of 1.51% based on the **P12**:PCBM blend film. Research on phenyl-DPP-based polymers was limited in the amount just because of its large dihedral angles between the benzene ring and DPP core. Such nonplanar conformation, to some extent, limited the application of such polymers. To relieve the above-mentioned steric effect, the nitrogen atom was embedded in the flanking units. Jung and co-workers reported polymer **P13** based on pyridine-flanked DPP (PyDPP).^36^ Since the increased electron deficiency of PyDPP was induced by electron-withdrawing nitrogen atoms, **P13** showed lower HOMO/LUMO energy levels than those of thiophene- and phenyl-bridged DPP units (Fig. 3a). A PCE of 4.9% with a high open-circuit voltage (*V*_oc_) up to 0.92 V was achieved by the **P13**:PC_71_BM blend film. The PCE value was much higher than that of **P12**. On account of the alleviated repulsive interaction and improved π-conjugation of the molecular backbone owing to the intramolecular N⋯H hydrogen bond, **P13**-based devices also exhibited excellent ambipolar transport performance with the max hole and electron mobilities of 2.78 and 6.30 cm^2^ V^−1^ s^−1^, respectively.^37^ Such preferable performance was attributed to the π-spacing distance of 3.60 for **P13**, which was much shorter than that of its thiophene-based counter polymer, thereby favoring the intermolecular hopping of the charge carrier and eventually achieving higher mobilities. More recently, Liu and co-workers synthesized another pyridine-DPP-based polymer **P14**, possessing deep-lying FMO energy levels.^38^ The resulting easier electron injection and increased hole carrier injection barrier of **P14** successfully led to its electron dominant transport performance with a *μ*_e_ of 2.22 cm^2^ V^−1^ s^−1^. Compared with the pyridine unit, the pyrazine unit has two nitrogen atoms substituted on the *para*-position of the benzene ring, which could further draw down the FMO energy levels of the polymer, *i.e.*, **P15**, which was constructed on the pyrazine-bridged DPP (PzDPP) unit.^39^ Apart from the much-lowered FMO energy levels, **P15** featured a suppressed backbone distortion due to the formed multiple hydrogen bonds. Unlike other DPP derivatives, most of which showed hole dominant ambipolar transport performances, **P15** exhibited unipolar n-type performance with its electron mobility reaching 1.67 cm^2^ V^−1^ s^−1^. Moreover, they tested the electrical conductivity of **P15** and gave a high value of 8.4 S cm^−1^ with its power factor reaching 57.3 μW m^−1^ K^−2^. Considering that the electrical conductivities of most n-doped polymers were around 1 S cm^−1^ with their power factors of no more than 10 μW m^−1^ K^−2^, the pyrazine-based polymer **P15** exhibited an impressive enhancement. The nitrogen embedding strategy was not only applied in the benzene ring, the thiophene unit could be similarly modified as well. The thiazole-based DPP in polymer **P16** helped achieve electron-dominant performance by adjusting its FMO energy levels and interchain packing.^40^ Besides, the **P16**/PDPP5T blend film also afforded a PCE of 2.9%. Such a PCE value was improved by another thiazole-DPP-based polymer **P17**, which had a low photon energy loss, thus exhibiting a PCE up to 5.6%, with a low energy loss of less than 0.6 eV after blending with PC_71_BM.^41^ Shortly afterward, Chen and co-workers synthesized three polymers (**P18**, **P19**, and **P20**) with different side chains.^42^ The pyridine-flanked DPP could address the limitation of large dihedral angles and provided a more electron-deficient condition. Therefore, high and balanced ambipolar charge transport properties were achieved by **P18-P20**, whose highest electron and hole mobilities reached 1.14 and 1.46 cm^2^ V^−1^ s^−1^, respectively. As described above, all the bridged moieties of DPP units are aromatic single rings. Besides, fused rings with extended conjugation length provided the polymer rigid backbone with better planarity and delocalized FMO distribution along the polymer main chain. Bronstein and co-workers extended the thiophene flanking unit to thieno[3,2-*b*]thiophene to increase the intermolecular association and synthesized the **P21**.^43^ Such extension contributed to the evenly distributed HOMO energy level and facilitated intermolecular charge carrier hopping. Eventually, ambipolar performance was achieved, with hole and electron mobilities of 1.95 and 0.07 cm^2^ V^−1^ s^−1^, respectively. And the OSC device could also afford a PCE of 5.4%. To further increase the polymer electron affinity (EA) and reduce the steric hindrance between the flanking unit and DPP core, polymer **P22** was synthesized with the thieno[2,3-*b*]pyridine-bridged DPP (TPDPP) unit.^44^ The single-crystal structure of the TPDPP molecule substituted with two short 2-ethylhexyl side chains showed a small dihedral angle of 7.1° between the core and bridges, which facilitated the appropriate planarity of **P22** (Fig. 3b). Such a planar molecular backbone ensured good main-chain conjugation and the delocalization of π-electrons. The **P22**/PTB7-Th blend film showed a PCE value of 2.72% with the open-circuit voltage up to 1.04 V. With the replacement of thiophene by a benzene ring, **P23** and **P24** were synthesized based on the more electron-deficient quinoline-flanked DPP monomer (QDPP).^45^ The additional fused benzene ring provided **P23** and **P24** with extended π-conjugation lengths and shorter π–π stacking distances, which invited ambipolar transport performances for both the polymers. The flexible FET devices on PET substrates (Fig. 3c) of **P23** exhibited balanced hole/electron mobilities of around 0.5–0.7 cm^2^ V^−1^ s^−1^. For polymer **P24**, the electron mobility reached 6.04 cm^2^ V^−1^ s^−1^ (Table 1). Moreover, the ambipolar mobilities of **P23** and **P24** were much higher than those of their pyridine-based counter polymers (Fig. 3d).

**Table 1:** Summary of physical and chemical properties and device performances for polymers **P1–P191**

Polymers	UV-vis *λ*^sol^_max_/*λ*^film^_max_ (nm)	HOMO/LUMO^a^ (eV)	Band Gap (eV) *E*^opt^_g_/*E*^ec^_g_	*μ*_h_/*μ*_e_ (cm^2^ V^−1^ s^−1^)	Max PCE (%)	Device configuration OFET (OSCs)	Ref.
**P1**	—	−5.17/−3.61	1.40/1.56	0.04/0.01	4.70	BGBC	26
**P2**	838/857	−5.07/—	—	3.10/—	—	TGBC	27
**P3**	—	—	—	2.1(26.2^b^)/—	—	BGBC	28
**P4**	—/789	−5.40/−3.80	1.41/1.60	—	3.80(5.00^b^)	(ITO/PEDOT:PSS/polymer:PCBM/LiF/Al)	29
**P5**	—/767	−5.50/−3.80	1.35/1.70	—	3.00(4.10^b^)	(ITO/PEDOT:PSS/polymer:PCBM/LiF/Al)	29
**P6**	—	—	1.59/1.66	3 × 10^−3^/7 × 10^−3^	3.70	BGBC	30
**P7**	782/782	−5.36/—	1.37/—	3.56/—	—	BGTC	31
**P8**	—/796	−5.19/−3.63	1.56/—	—	4.56	(ITO/PEDOT:PSS/polymer:PCBM/LiF/Al)	32
**P9**	827/882	−5.20/−4.02	1.18/—	0.10/0.10	—	BGBC	33
**P10**	823/864	−5.10/−3.92	1.21/—	0.26/0.05	—	BGBC	33
**P11**	790/836	−5.30/−3.97	1.33/—	0.95(1.51^b^)/—	—	BGTC	34
**P12**	539/563	−5.47/−3.74	1.70/1.73	—	1.51	(ITO/polymer:PCBM/Al)	35
**P13**	664/684	−5.77/−3.86	1.71/1.91	—	4.90	(ITO/PEDOT:PSS/polymer:PCBM/Ca/Al)	36
**P14**	654/672	−5.75/−4.03	1.72/—	0.58/2.22	—	TGBC	38
**P15**	713/—	−5.89/−4.03	1.86/—	—/1.67	—	TGBC	39
**P16**	—	−5.63/−4.00	1.44/1.63	6 × 10^−3^/0.13	2.90	TGBC (ITO/ZnO/PDPP5T:polymer/MoO3/Ag)	40
**P17**	—	−4.78/−3.11	1.28/1.67	—	5.60	(ITO/MoO_3_/active layer/LiF/Al)	41
**P18**	770/702	−5.73/−3.66	1.42/2.07	0.77/0.497	—	TGBC	42
**P19**	770/776	−5.80/−3.72	1.39/2.08	1.46/1.14	—	TGBC	42
**P20**	770/702	−5.81/−3.72	1.41/2.09	0.489/0.202	—	TGBC	42
**P21**	812/746	−5.06/−3.68	1.37/—	1.95/0.03	5.40	TGBC (ITO/PEDOT/polymer:PCBM/LiF/Al)	43
**P22**	711/—	—	—	—/0.1	2.72	TGBC	44
**P23**	—	−5.42/−3.68	1.74/—	0.5/0.72	—	TGBC	45
**P24**	—	−5.64/−3.84	1.80/—	0.21/6.04	—	TGBC	45
**P25**	—	−4.80/−3.49	1.36/1.31	5.87/—	6.10	BGBC	46
**P26**	—	−4.80/−3.49	1.34/1.31	12.5/—	6.50	BGBC	46
**P27**	780/785	−5.48/−4.03	1.45/—	3.05/—	5.90	BGBC (ITO/ZnO/polymer:PCBM/MoO3/Ag)	47
**P28**	774/774	−5.36/−3.93	1.43/—	0.32/—	5.70	BGBC (ITO/ZnO/polymer:PCBM/MoO3/Ag)	47
**P29**	—	−5.28/−3.55	1.53/1.73	0.18/0.48	5.48	TGBC (ITO/ZnO/polymer:PCBM/MoOx/Ag)	48
**P30**	—	−5.14/−3.50	1.54/1.64	0.55/0.08	7.56	TGBC (ITO/ZnO/polymer:PCBM/MoOx/Ag)	48
**P31**	—	−4.73/−3.24	1.49/—	1.20/0.40	5.59	TGBC (ITO/ZnO/polymer:PCBM/MoOx/Ag)	49
**P32**	—	−5.09/−3.63	1.46/—	1.68/0.14	6.96	TGBC (ITO/ZnO/polymer:PCBM/MoOx/Ag)	49
**P33**	—	−5.29/−3.83	1.46/—	1.50/0.35	5.62	TGBC (ITO/ZnO/polymer:PCBM/MoOx/Ag)	49
**P34**	831/833	−5.24/−3.91	1.17/1.33	0.074/0.053	—	TGBC	50
**P35**	832/833	−5.29/−3.85	1.18/1.44	0.48/0.12	—	TGBC	50
**P36**	832/832	−5.27/−3.74	1.19/1.53	1.09/0.25	—	TGBC	50
**P37**	833/833	−5.24/−3.73	1.16/1.51	0.76/0.13	—	TGBC	50
**P38**	796/806	−5.33/−3.79	1.20/1.54	0.86/0.44	—	TGBC	50
**P39**	785/782	−5.29/−4.21	—/1.08	0.021/1.2 × 10^−4^	—	TGBC	50
**P40**	—	−5.06/−3.68	1.44/—	0.12/—	—	BGBC	51
**P41**	—	−5.16/−3.20	1.49/1.96	—	5.90	(ITO/MoO_3_/polymer:PCBM/LiF/Al)	59
**P42**	—	−5.19/−3.20	1.49/1.99	—	6.60	(ITO/MoO_3_/polymer:PCBM/LiF/Al)	59
**P43**	569/642	−5.51/−3.52	1.59/1.99	0.034/—	5.10	BGTC (ITO/PEDOT:PSS/polymer:PCBM/LiF/Al)	62
**P44**	785/825	−5.19/−3.50	1.27/2.09	0.02/0.02	—	BGBC	64
**P45**	524/538	−5.39/−3.35	1.90/2.04	—/1 × 10^−3^	—	BGBC	65
**P46**	579/579	−5.25/−3.50	1.68/1.75	1 × 10^−3^/1 × 10^−3^	—	BGBC	65
**P47**	615/639	−5.68/−3.96	1.69/1.72	0.21/0.18	—	TGBC	66
**P48**	788/806	−5.49/−4.17	1.32/—	0.053/0.021	—	BGBC	67
**P49**	615/678	−5.37/−3.96	1.38/1.41	—	—	—	68
**P50**	687/712	−5.66/−4.20	1.33/1.46	0.069/0.667	—	BGBC	69
**P51**	675/703	−5.78/−4.31	1.39/1.47	0.021/0.227	—	BGBC	69
**P52**	—/665	−5.56/−4.08	1.46/1.48	—/0.01	—	BGBC	70
**P53**	834/858	−5.00/−3.70	1.10/1.30	1.24/0.82	—	BGBC	72
**P54**	790/815	−5.00/−3.60	1.20/1.40	1.37/—	—	BGBC	72
**P55**	789/798	−5.28/−3.79	1.25/1.49	1.08/2.23	—	TGBC	76
**P56**	628/624	−5.58/−3.87	1.71/—	—	7.30	(ITO/ZnO/PEOz/PBFTAZ:polymer/MoO_3_/Ag)	79
**P57**	—/645	−5.82/−3.83	1.50/—	—	6.30	(ITO/PEDOT:PSS/polymer: PCBM/LiF/Al)	80
**P58**	703/709	−5.69/−3.82	1.61/1.59	—	10.7	(ITO/ZnO/PEIE/polymer:PCBM/MoO3/Ag)	81
**P59**	728/729	−5.54/−3.70	1.53/1.84	3.49/2.77	—	TGBC	82
**P60**	686/689	−5.82/−3.83	1.62/1.99	3.94/3.50	—	TGBC	82
**P61**	711/720	−5.66/−3.46	1.54/2.20	5.33/2.06	—	TGBC	83
**P62**	718/720	−5.63/−3.55	1.57/2.08	6.41/6.76	—	TGBC	83
**P63**	726/731	−5.72/−3.64	1.40/2.08	2.75/9.70	—	TGBC	83
**P64**	742/756	−5.67/−3.64	1.50/2.03	7.28/6.43	—	BGBC	84
**P65**	1050/1035	−4.86/−3.73	0.92/1.13	0.16/0.14	—	BGBC	85
**P66**	927/925	−4.84/−3.46	1.04/1.38	0.39/—	—	BGTC	86
**P67**	880/868	−4.98/−3.53	1.15/1.45	0.45/—	—	BGTC	86
**P68**	730/759	−5.12/−3.49	1.36/1.63	14.4/—	—	TGBC	87
**P69**	848/839	−5.13/−3.58	1.21/1.55	3.93/1.07	—	TGBC	88
**P70**	914/926	−4.90/−3.90	1.05/1.00	0.40/0.70	—	TGBC	89
**P71**	897/909	−4.80/−3.70	1.13/1.10	0.20/0.20	—	TGBC	89
**P72**	864/875	−4.80/−3.60	1.19/1.20	0.40/0.10	—	TGBC	89
**P73**	—/665	−5.10/−3.50	1.60/—	0.31/—	9.1	(ITO/ZnO/polymer: PCBM/MoO_3_/Ag)	90
**P74**	670/665	−5.15/−3.67	1.48/—	—	5.0	(ITO/ZnO/polymer: PCBM/MoO_x_/Ag)	91
**P75**	642/640	−5.39/−3.80	1.59/—	—	5.6	(ITO/ZnO/polymer: PCBM/MoO_x_/Ag)	91
**P76**	660/665	−5.34/−3.86	1.58/—	—	5.1	(ITO/ZnO/polymer: PCBM/MoO_x_/Ag)	91
**P77**	—	−5.07/−3.51	1.56/—	—	6.2	(ITO/PEDOT:PSS/polymer:PCBM/Ca/Al)	92
**P78**	—	−5.10/−3.54	1.56/—	—	6.92	(ITO/PEDOT:PSS/polymer:PCBM/Ca/Al)	92
**P79**	729/729	−5.20/−3.67	1.53/—	—	9.63	(ITO/PEDOT:PSS/polymer:PCBM/Ca/Al)	92
**P80**	505/501	—	2.32/—	—	5.75	(ITO/PEDOT:PSS/polymer:PCBM/Ca/Al)	93
**P81**	523/523	—	2.23/—	—	6.32	(ITO/PEDOT:PSS/polymer:PCBM/Ca/Al)	93
**P82**	551/575	—	2.16/—	0.55/—	—	BGBC	94
**P83**	505/541	—	2.29/—	6 × 10^−3^/—	—	BGBC	94
**P84**	—	−5.42/−3.57	—/1.85	—	11.0	(ITO/PEDOT:PSS/polymer:m-ITIC/PDINO/Al)	96
**P85**	781/801	−5.54/−3.84	1.37/1.70	8.8 × 10^−2^/—	3.39	BGTC (ITO/PEDOT:PSS/polymer:PCBM/LiF/Al)	97
**P86**	737/773	−5.30/−3.72	1.39/1.58	0.18/—	4.79	BGTC (ITO/PEDOT:PSS/polymer:PCBM/LiF/Al)	97
**P87**	673/668	−5.34/−3.62	1.55/1.71	8.95 × 10^−4^/—	5.03	BGBC (ITO/PEDOT:PSS/polymer:PCBM/Ca/Al)	99
**P88**	663/677	−5.77/−3.66	2.03/2.11	—	3.12(5.26^b^)	(ITO/PFN/polymer:PCBM/MoO_3_/Ag)	100
**P89**	708/706	−5.24/−3.56	1.44/1.68	4.82/1.11	—	TGBC	101
**P90**	706/703	−5.11/−3.55	1.45/1.56	8.09/0.74	—	TGBC	101
**P91**	594/606	−5.24/−3.72	1.71/1.52	—	3.49(6.41^b^)	(ITO/PEDOT:PSS/polymer:PCBM/LiF/Al)	102
**P92**	600/606	−5.41/−3.34	1.72/2.07	0.35/—	—	BGBC	103
**P93**	600/626	−5.40/−3.62	1.68/1.78	0.037/—	—	BGBC	103
**P94**	628/628	−5.25/−3.36	1.63/1.89	0.33/—	—	BGBC	104
**P95**	624/630	−5.35/−3.39	1.64/1.96	0.39/—	—	BGBC	104
**P96**	624/632	−5.20/−3.36	1.60/1.84	0.30/—	—	BGBC	104
**P97**	616/628	−5.27/−3.43	1.63/1.84	0.48/—	—	BGBC	104
**P98**	646/648	−5.82/−3.89	1.58/1.93	0.47/—	—	BGBC	104
**P99**	632/642	−5.99/−3.93	1.60/2.06	0.31/—	—	BGBC	104
**P100**	944/986	−5.33/−3.86	0.95/1.47	0.16/0.14	—	BGBC	105
**P101**	—	−5.70/−3.88	1.42/1.82	1.70/1.37	—	TGBC	106
**P102**	1107/1107	−5.18/−3.94	0.71/1.24	0.45/0.22	—	TGBC	107
**P103**	—	−5.72/−4.15	1.31/1.67	—/1.74	—	TGBC	108
**P104**	—	−5.80/−4.37	1.32/1.43	—/3.22	—	TGBC	108
**P105**	812/876	−5.87/−3.92	1.26/1.95	5.97/7.07	—	TGBC	109
**P106**	838/844	−5.96/−4.32	1.31/1.64	—/1.24	—	TGBC	110
**P107**	806/794	−5.60/−4.06	1.23/1.54	0.10/0.14	—	BGBC	111
**P108**	787/777	−5.76/−3.79	1.45/1.97	0.12/0.14	—	BGBC	112
**P109**	816/806	−5.65/−3.84	1.41/1.81	0.51/0.50	—	BGBC	112
**P110**	844/845	−5.68/−3.77	1.31/1.91	0.15/0.33	—	BGBC	113
**P111**	699/699	−5.60/−3.71	1.23/1.89	0.19/0.088	—	BGBC	114
**P112**	711/698	−5.71/−3.70	1.22/2.01	0.10/0.075	—	BGBC	114
**P113**	—	−5.32/−4.05	1.27/—	1.92/—	—	BGBC	115
**P114**	787/787	−5.16/−3.58	1.31/1.58	1.55/0.021	—	BGTC	116
**P115**	788/781	−5.24/−3.58	1.29/1.67	1.79/0.087	—	BGTC	116
**P116**	—	−5.47/−3.79	1.55/1.68	0.055/0.069	—	TGBC	117
**P117**	—	−5.39/−3.77	1.52/1.62	0.10/0.14	—	TGBC	117
**P118**	—/850	−5.40/−4.40	1.01/—	—/10^−5^	—	BGTC	119
**P119**	—/927	−5.20/−4.20	1.01/—	—/0.03	—	BGTC	119
**P120**	—/1128	−5.00/−4.20	0.84/—	—/0.001	—	BGTC	119
**P121**	690/—	−5.99/−4.49	0.89/1.50	—/0.18	—	—	120
**P122**	693/707	−5.25/−3.77	1.48/—	—/0.85	—	TGBC	122
**P123**	384/390	−5.55/−3.91	1.63/1.64	—	9.42	(ITO/PEDOT:PSS/PffBT4T-2OD:PCBM/polymer/Ag)	125
**P124**	385/391	−5.47/−4.18	1.56/1.29	—	10.1	(ITO/PEDOT:PSS/PffBT4T-2OD:PCBM/polymer/Ag)	125
**P125**	713/698	−5.84/−3.81	—/2.03	1.70/8.50	—	TGBC	127
**P126**	666/664	−6.20/−3.88	—/2.32	—/3.50	—	TGBC	127
**P127**	682/683	−5.90/−3.77	—/2.13	0.70/3.10	—	TGBC	127
**P128**	673/669	−6.24/−3.85	—/2.39	—/2.20	—	TGBC	127
**P129**	—/676	−5.71/−3.80	1.50/1.91	0.003/3.95	—	TGBC	128
**P130**	—/917	−5.40/−3.87	1.08/1.53	0.008/7.37	—	TGBC	128
**P131**	**—**	−5.60/−4.40	1.20/—	0.10/0.27	—	BGTC	129
**P132**	833/814	−5.60/−4.00	—/1.60	—	5.57	(ITO/PEDOT: PSS/BDDT:polymer/Ca/Al)	130
**P133**	—	−6.00/−4.20	—/1.80	—/5.60 × 10^−3^	—	BGTC	131
**P134**	—	−6.05/−4.22	—/1.83	—/0.12	—	BGTC	131
**P135**	—	−6.05/−4.22	—/1.83	—/0.55	—	BGTC	131
**P136**	500/515	−5.41/−3.29	2.00/2.12	—	5.04	(ITO/PEDOT:PSS/polymer:PCBM/Ca/Al)	132
**P137**	502/518	−5.47/−3.46	1.98/2.01	—	6.35	(ITO/PEDOT:PSS/polymer:PCBM/Ca/Al)	132
**P138**	607/610	−6.05/−4.01	1.71/2.04	—/0.11	7.52	BGTC (ITO/ZnO/PTB7−Th:polymer/MoO_3_/Ag)	133
**P139**	638/640	−5.76/−3.96	1.59/1.80	8.70 × 10^−4^/1.79 × 10^−3^	—	BGTC	133
**P140**	440/444	−5.40/−3.88	1.06/1.52	0.02/—	—	BGBC	134
**P141**	524/—	−5.99/−3.33	2.07/2.66	—/0.039	—	TGBC	135
**P142**	551/—	−5.76/−3.27	1.94/2.49	—/0.029	—	TGBC	135
**P143**	592/—	−5.85/−3.38	1.87/2.47	—/0.19	—	TGBC	135
**P144**	—/562	−5.90/−3.96	1.74/1.94	—	6.85	(ITO/PEDOT:PSS/PBDT-TS1:polymer/Mg/Al)	137
**P145**	427/432	−5.97/−3.97	1.74/2.00	—	3.52	(ITO/PEDOT:PSS/PTB7-Th:polymer/Ca/Al)	138
**P146**	407/410	−6.18/−3.95	1.86/2.23	—	0.97	(ITO/PEDOT:PSS/PTB7-Th:polymer/Ca/Al)	138
**P147**	408/413	−5.73/−4.04	1.56/1.69	—	1.43	(ITO/PEDOT:PSS/PTB7-Th:polymer/Ca/Al)	138
**P148**	444/625	−5.56/−3.70	1.86/—	0.04/0.30	—	TGBC	139
**P150**	498/755	−5.01/−3.70	1.31/—	3 × 10^−3^/0.03	—	TGBC	139
**P151**	380/—	−6.00/−3.80	1.79/2.20	—	4.70	(ITO/PEDOT:PSS/PTB7-Th:polymer/Ca/Al)	140
**P152**	—	−5.82/−4.12	1.70/—	—	6.39	(ITO/PEDOT:PSS/PTB7-Th:polymer/Au)	141
**P153**	—	−5.94/−4.15	1.79/—	—	5.35	(ITO/PEDOT:PSS/PTB7-Th:polymer/Au)	141
**P154**	402/547	−5.94/−4.03	1.91/—	—	8.59	(ITO/ZnO/PTB7-Th:polymer/V_2_O_5_/Al)	142
**P155**	—	−5.45/−3.80	1.29/1.65	—/0.30	—	BGTC	144
**P156**	—	−5.14/−3.74	0.94/1.40	—/0.09	—	BGTC	144
**P157**	—	—	—	—	0.03	(ITO/PEDOT:PSS/polymer/Al)	145
**P158**	—	—	—	—	0.03	(ITO/PEDOT:PSS/polymer/Al)	145
**P159**	491/507	−5.39/−3.36	2.03/—	—	9.48	(ITO/PEDOT:PSS/polymer:IDIC/Au)	147
**P160**	510/516	−5.25/−3.25	2.00/—	—	8.31	(ITO/PEDOT:PSS/polymer:IDIC/Au)	147
**P161**	575/583	−5.55/−3.80	1.75/—	0.63(0.21^c^)/—	13.3	TGBC (ITO/PEDOT:PSS/polymer:IT-4F/PDINO/Al)	148
**P162**	580/580	−5.63/−3.86	1.77/—	0.93(0.23^c^)/—	12.7	TGBC (ITO/PEDOT:PSS/polymer:IT-4F/PDINO/Al)	148
**P163**	551/587	−5.34/−3.46	1.81/—	—	8.63	(ITO/PEDOT:PSS/polymer:PCBM/PFN-Br/Al)	149
**P164**	545/608	−5.90/−3.60	1.80/2.30	—	10.2	(ITO/PEDOT:PSS/polymer:ITIC/PDINO/Al)	150
**P165**	578/598	−5.97/−3.63	1.81/2.34	—	12.1	(ITO/PEDOT:PSS/polymer:ITIC/PDINO/Al)	150
**P166**	—	−5.56/−3.75	1.80/1.81	—	9.30	(ITO/PEDOT:PSS/polymer:PBDTTT-C-T:PCBM/Ca/Al)	151
**P167**	518/567	−5.65/−3.90	1.75/—	—	9.21	(ITO/ZnO/polymer:PCBM/MoO_3_/Ag)	153
**P168**	533/593	−5.39/−3.59	1.80/—	0.74/—	—	TGBC	154
**P169**	480/534	−5.67−/3.74	1.93/—	0.32/—	—	TGBC	154
**P170**	610/630	−5.20/−3.45	1.78/1.75	—	14.2	(ITO/PEDOT:PSS/polymer:Y6/PFN-Br/Ag)	155
**P171**	—/578	−6.18/−4.05	—/2.13	—/0.06	—	BGTC	156
**P172**	—/535	−6.15/−4.00	—/2.15	—/2.55	—	BGTC	156
**P173**	—/641	−5.48/−3.17	1.78/—	0.06/—	5.60	BGTC (ITO/ZnO/polymer:PCBM/MoOx/Ag)	158
**P174**	—/660	−5.49/−3.25	1.71/—	0.05/—	8.00	BGTC (ITO/ZnO/polymer:PCBM/MoOx/Ag)	158
**P175**	—/654	−5.62/−3.20	1.73/—	0.03/0.02	2.40	BGTC (ITO/ZnO/polymer:PCBM/MoOx/Ag)	158
**P176**	—/602	−6.02/−3.36	1.91/—	—/0.05	0.05	BGTC (ITO/ZnO/polymer:PCBM/MoOx/Ag)	158
**P177**	587/588	−5.43/−3.45	1.98/—	—/3.10	6.67	TGBC (ITO/PEDOT:PSS/PTB7-Th:polymer/LiF/Al)	159
**P178**	606/616	−5.43/−3.55	1.88/—	—/1.23	8.61	TGBC (ITO/PEDOT:PSS/PTB7-Th:polymer/LiF/Al)	159
**P179**	—	−5.27/−3.43	1.84/—	—/1.13	6.85	TGBC (ITO/PEDOT:PSS/PTB7-Th:polymer/ETL/Al)	160
**P180**	—	−5.28/−3.30	1.98/—	—/0.84	0.03	TGBC (ITO/PEDOT:PSS/PTB7-Th:polymer/ETL/Al)	160
**P181**	—/588	−6.08/−3.50	1.96/—	—/2.73	6.50	TGBC (ITO/PEDOT:PSS/PTB7-Th:polymer/LiF/Al)	161
**P182**	—	−5.94/−3.46	1.74/2.48	—	8.10	(ITO/PEDOT:PSS/active layer/LiF/Al)	162
**P183**	—	−5.78/−3.77	2.01/—	—/1.61	—	TGBC	163
**P184**	—	−5.97/−4.09	1.88/—	—	19.5	PVSCs (ITO/PTAA/perovskite/polymer/BCP/Ag)	164
**P185**	—	−5.79/−4.01	1.78/—	—	20.8	PVSCs (ITO/PTAA/perovskite/polymer/BCP/Ag)	164
**P186**	628/666	−5.44/−3.05	1.65/2.39	—	8.61	(ITO/ZnO/polymer:PCBM/MoO_3_/Ag)	165
**P187**	618/658	−5.41/−3.01	1.67/2.40	—	10.2	(ITO/ZnO/polymer:PCBM/MoO_3_/Ag)	165
**P188**	590/635	−5.48/−2.99	1.72/2.49	—	8.47	(ITO/ZnO/polymer:PCBM/MoO_3_/Ag)	165
**P189**	643/630	−5.59/−3.66	1.60/1.93	1.17 × 10^−2^/2.78 × 10^−3^	—	TGBC	166
**P190**	594/585	−5.85/−3.75	1.62/2.10	1.38 × 10^−1^/7.39 × 10^−2^	—	TGBC	166
**P191**	560/587	−5.70/−3.68	1.67/2.02	7.42 × 10^−3^/8.04 × 10^−4^	—	TGBC	166

a The frontier energy levels calculated from the cyclic voltammetry or UPS.

b The performance of the polymer having additives.

c The mobilities extracted in the linear region.

#### 2.1.2. Polymers with asymmetrically flanked DPP units

The above polymers are based on symmetrically flanked DPP units and corresponding derivatives. Also, DPP derivatives bridged by two different units showed different physicochemical properties, for example, molecular dipole moments. The larger dipole moments of asymmetrically flanked DPP units and concerned polymers induce their different solution processabilities in polar and non-polar organic solvents, thereby leading to varied performances in optoelectronics. Chlorine-containing solvents, like chloroform (CHCl_3_), chlorobenzene (CB), and *ortho*-dichlorobenzene (*o*-DCB), were common processing solvents. Those solvents could adversely affect the human body and the environment. To develop nonchlorinated-solvent friendly polymers, Ji and co-workers synthesized two polymers, namely **P25** and **P26**, in which the DPP core was asymmetrically substituted with thiophene and thieno[3,2-*b*]thiophene units.^46^ The destroyed mainchain symmetry gave a good solubility of **P25** and **P26** in toluene. Their films processed from the mixed solvent (toluene and diphenyl ether) afforded a high hole mobility of 12.5 cm^2^ V^−1^ s^−1^. Moreover, the processed **P25**:PC_71_BM and **P26**:PC_71_BM films exhibited an enhanced PCE of 6.1% and 6.5%, respectively (Table 1).

In addition to substituting the DPP core with dual electron donors, researchers also tried to replace one of the flanking rings with the electron-withdrawing unit, thus adjusting the electron deficiency of building blocks. For instance, the structures of **P27** and **P28** were built up with the thiophene/thiazole-flanked DPP units in asymmetric and random ways, respectively.^47^ In comparison with **P28**, the ordered structure of **P27** afforded better planarity with smaller dihedral angles, thereby forming a larger domain size and adopting more percentage of favorable face-on orientation in its film. Those properties provided **P27** with a relatively higher hole mobility of 3.05 cm^2^ V^−1^ s^−1^ and a higher PCE of 5.90% than those of **P28**. Just like the thiazole unit, pyridine is another available electron-deficient aromatic ring that can be used as the linker. Qiu and co-workers asymmetrically substituted DPP units with thiophene and pyridine rings in polymers **P29** and **P30**.^48^ Owing to the more electron-deficient pyridine units, **P29** and **P30** showed deep-lying HOMO energy levels and narrow bandgaps, which led to the promising hole/electron mobilities of 0.18/0.48 and 0.08/0.55 cm^2^ V^−1^ s^−1^, respectively. In agreement with their wide absorption spectra, the **P29**:PC_71_BM and **P30**:PC_71_BM gave their respective PCE of 5.48% and 7.56%, both of which were higher than those of their pyridine-DPP-based analogs. To further substitute π-extended aromatic rings in DPP units, thiophene/benzothiophene flanked DPP was used in polymers **P31-P33**.^49^ Such changes not only widened their absorption bands but also simultaneously improved their *J*_sc_ and PCE values. **P32** afforded the highest PCE of 7.0% among the three polymers. The PCEs of the other two polymers also achieved 5.6%. Moreover, the electron/hole mobilities of **P31**, **P32**, and **P33** were 1.20/0.40 cm^2^ V^−1^ s^−1^, 1.68/0.14 cm^2^ V^−1^ s^−1^, and 1.50/0.35 cm^2^ V^−1^ s^−1^, respectively. Benefitting from the extended π-conjugation and delocalized orbitals, both hole and electron mobilities got improved, especially the electron mobility, which was almost four times higher than that of the PyTDPP-based counterpart. The DPP flanked by π-extended aromatic rings was proved to be preferable for application in both FETs and OSCs, which facilitated more studies on this strategy. Song and co-workers reported two novel DPP derivative units, DDPP-PhCO and DDPP-PhCN, in which two thiophene-based DPP units were combined with polycyclic aromatics.^50^ DDPP-PhCO-based **P34-P38** and DDPP-PhCN-based **P39** exhibited much-widened absorption spectra due to their extended π-conjugation. Despite the limited adjustment of LUMO energy levels, additional cyano and carbonyl groups induced the polymer films to have orderly packing, resulting in ambipolar transport characteristics with the max hole and electron mobilities reaching 1.09 and 0.44 cm^2^ V^−1^ s^−1^, respectively.

**Fig. 4:**
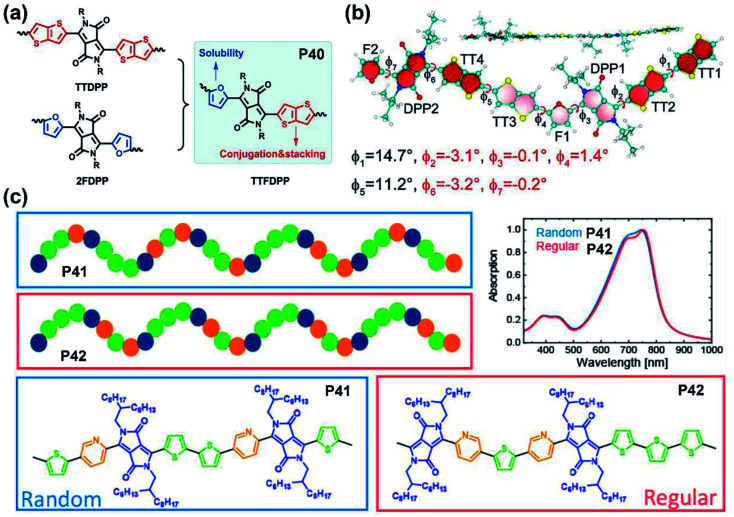
(a) The synthetic strategy of **P40** to combine the advantages of two other polymers. Reproduced from ref. 51 with permission from Wiley-VCH, copyright 2018. (b) Optimized structure of the **P40** dimer with the dihedral angles marked. Reproduced from ref. 51 with permission from Wiley-VCH, copyright 2018. (c) The illustration of backbone shapes and the UV-vis absorption spectra of **P41** and **P42**. Reproduced from ref. 59 with permission from the American Chemical Society, copyright 2020.

As mentioned in the previous section and other related studies, furan-DPP-containing polymers performed well in their solubilities, carrier transport behaviors, and OSC device performance. Therefore, the furan-containing asymmetrical DPP building block was inserted into the polymer backbone for better solution processability. The furan/thieno[3,2-*b*]thiophene-DPP (TTFDPP)-based polymer **P40** combined the advantages of two flanking units together and achieved an improved solubility, self-assembly ability, and more conjugated molecular backbone (Fig. 4a).^51^ The DFT simulation revealed a planar backbone, with dihedral angles no more than 3.2° in the TTFDPP moiety (Fig. 4b), and delocalized HOMO/LUMO orbitals over the whole molecular backbone. Owing to the enhanced solubility, the **P40** film was processed from mixed chloroform and toluene solvents, with its FET device exhibiting typical p-type performance.

As the performance regulator of the corresponding polymers, the different DPP flanking units could firstly adjust the planarity and decrease the backbone distortion through the intramolecular interactions and hydrogen bonds in the lactam structure.^52^ Secondly, some flanking units like furan are another factor that could positively affect the processing solubility of polymers, besides the lactam nitrogen alkylation.^53^ This function directly leads to the feasibility of obtaining polymers with shorter sidechain substitution, which is conducive to the decreased lamellar *d*-spacing distance, increased crystallinity, and the ordered intermolecular packing state of polymer films.^54,55^ Finally, the incorporation of electron-deficient flanking units could tune FMOs to the lower level, which is pivotal to the carrier injection from the metal electrode to the semiconducting layer.^56^ Compared with polymers containing symmetrically flanked DPP units, both superiorities and defects existed in asymmetrically bridged DPP-based polymers. For one thing, the substitution of two different aromatic rings of the DPP unit could adjust the polymer in two or more aspects simultaneously, such as backbone planarity, conjugation length, solubility, *etc.*^57^ For another thing, the above properties could be fine-tuned by asymmetrical substitution. Nevertheless, asymmetrically flanked DPP units were combined with donors randomly, which results in incomparable symmetry and regularity of their molecular backbones to their symmetric counterparts. Such changes could make polymer films adopt different packing states, let alone their photoelectric performances.^57,58^ Besides, compared with DPPs with the same flanking rings, those asymmetrically bridged DPP units are relatively harder to obtain due to the multiple synthetic steps, low yield, and difficulties in the purification process. It is noted that the backbone randomness of asymmetrically flanked DPP-based polymers should not be neglected, which potentially affected the physicochemical properties or device performance. More recently, Leenaers and co-workers reported two polymers containing the thiophene/pyridine asymmetrically flanked DPP unit, **P41** and **P42**, containing a regiorandom and regioregular molecular backbone, respectively (Fig. 4c).^59^ Despite their similar optical properties, the regioregular polymer **P42** gave a relatively higher PCE of 6.6% than 5.9% of regioregular polymer **P41** (Table 1). Overall, the asymmetrical substitution strategy can effectively tune the polymer properties if the reaction sites of the copolymerization are controllable, which is also a future challenge.

#### 2.1.3. Polymers with modified DPP cores

The above modification strategies are focused on the adjacent flanking rings of DPP and its derivatives. The polymer properties can be effectively adjusted *via* changing two bridges in symmetric and asymmetric ways. Furthermore, the structural adjustment of the DPP core is equally efficacious in improving molecular conjugation and electron-withdrawing capacity.^60^ Therefore, extensive efforts have been dedicated to optimizing the molecular structure of the DPP core.

As the most structurally similar DPP-analog, pyrrolo[3,2-*b*]pyrrole-2,5-dione (isoDPP), with less synthetic complexity, has the position of the nitrogen atom and carbonyl group opposite to the DPP core. Song and co-workers synthesized a thiophene-bridged isoDPP with the amide nitrogen atoms substituted by butyl chain alkylated phenyl groups.^61^ Besides the p-type carrier transport performance, a PCE of 2.0% in photovoltaic cells without thermal annealing treatment was also achieved. Both performances were further improved by another isoDPP-based polymer **P43**, which showed n-channel carrier transport behavior and afforded a high PCE value of 5.1% (Table 1).^62^

**Fig. 5:**
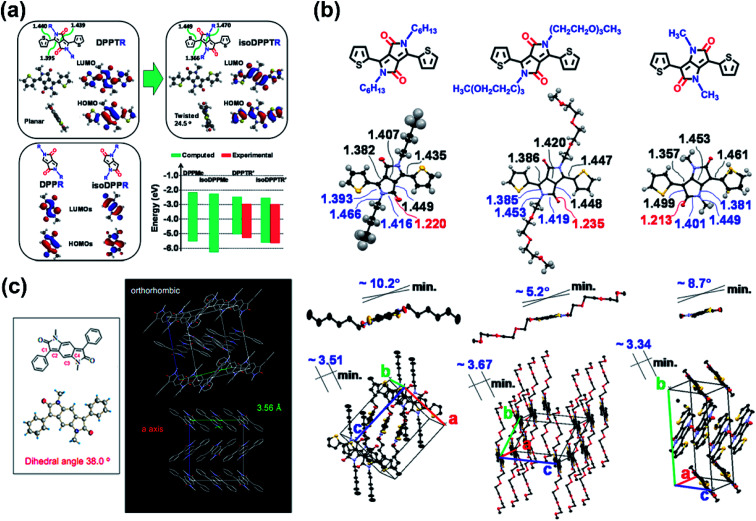
(a) The simulated structures of DPP and isoDPP monomers as well as the summary of their estimated and experimental HOMO/LUMO energy levels. Reproduced from ref. 62 with permission from the American Chemical Society, copyright 2013. (b) The chemical and crystal structures of two different chain substituted DPP units and the methyl-substituted isoDPP unit. Reproduced from ref. 62 with permission from the American Chemical Society, copyright 2013. (c) Single crystal structure of the building block of **P45–P46**. Reproduced from ref. 65 with permission from the American Chemical Society, copyright 2011.

The thiophene-isoDPP backbone was not comparable to that of thiophene-DPP, as indicated by their DFT-simulated dihedral angles which were 24.5° for thiophene-isoDPP and almost 0° for thiophene-DPP (Fig. 5a). Besides, the isoDPP-induced poor crystallinity was also revealed by another study.^63^ Those differences could be explained by their varied structures. Despite the simply exchanged positions of the carbonyl groups and nitrogen atoms in thiophene-DPP and thiophene-isoDPP, they had considerably different internal structures. Caused by the different conjugation ways, the length of the carbon–carbon double bonds in the isoDPP core was shorter than those in the DPP core and the adjacent single bonds elongated correspondingly (Fig. 5a and b), such changes directly lead to the more apparent steric effect between the flanking unit and the substituted sidechain, eventually resulting in the different backbone planarity and packing state. The structural disadvantage was inevitable but the adjustable structure and appropriate energy levels indicated the application potential of isoDPP nevertheless. For instance, in comparison with isoDPP- or DPP-based polymers, combining both the isoDPP and DPP moieties in one single polymer could potentially achieve a polymer film with better physicochemical properties and optoelectronic performance, *i.e.*, **P44**. The combination of thiophene-isoDPP and thiophene-DPP isomers provided the **P44** good film with aggregation and proper energy levels, leading to the balanced ambipolar transport behavior of **P44** with around 0.02 cm^2^ V^−1^ s^−1^ for both hole and electron mobilities.^64^ This performance was higher than those of its reference polymers based on only the DPP or isoDPP unit.

The easily changeable isoDPP structure could be modified by many methods. The extended isoDPP unit, benzodipyrrolidone (BDP), having two lactam structures fused with a benzene ring, was synthesized as early as 1980 as the dyestuff. Because of its DPP-similar structure and lactam moieties, the BDP moiety was deduced to be favorable for organic semiconductor materials. Two BDP-based polymers, **P45** and **P46**, were reported by Cui and co-workers.^65^ The single-crystal analysis of the methyl-substituted phenyl-BDP (BDPDP) molecule discovered the high planarity of the BDP core despite a torsion angle of 38° between the adjacent benzene ring and BDP core. The orthorhombic packing structure revealed the π–π interaction of the middle benzene ring and the benzene ring adjacent to the lactam structure with a distance of 3.56 Å (Fig. 5c). **P45** and **P46** exhibited n-type and ambipolar type behaviors, respectively, with the mobilities of around the magnitude of 10^−3^ cm^2^ V^−1^ s^−1^. The reasons for such properties could be complex, such as molecular weight, device architecture, the solvent used for the spin-coating process, and some other reasons. The structural novelty of BDP and its polymers attracted scientists to further improve its performance in electronics. Polymer **P47** was obtained and had the same structure as **P46** but with a higher number-averaged molecular weight (*M*_n_) of around 13.9 kDa (polydispersity: 5.20), which was higher than 8 kDa (polydispersity: 2.29) of **P46**.^66^ The higher molecular weight, together with device optimization, successfully enhanced its performance and gave it balanced electron and hole mobilities of 0.18 and 0.21 cm^2^ V^−1^ s^−1^, respectively, due to the reduced contact resistance. Additionally, the nitrogen embedded BDP building block (PzDP) was also designed and utilized in polymer **P48**, which had a deep-lying LUMO energy level of −4.17 eV.^67^ Besides, the existence of nitrogen atoms decreased the torsional angles between the benzene ring and the PzDP core, due to the N⋯H hydrogen bond. The spin-coated polymer **P48** film also exhibited ambipolar FET performance. Despite the moderate mobilities, such effective adjustment to the narrower bandgap was still worthy of being borrowed. To further extend the BDP unit, the naphthodipyrrolidones (NDP) unit, with one more benzene ring fused in the BDP unit, was chosen as the building block of **P49**.^68^ The single-crystal analysis of the phenyl-flanked NDP unit (PhNDP) revealed its intermolecularly slipped π–π stacking state, with a shorter distance of 3.38 Å. Moreover, the planar backbone of PhNDP, with a dihedral angle of 25°, led to broad absorption bands and a narrow bandgap of the **P49** film. The above characteristics indicated the easier carrier injection, ambipolar transport property, and excellent air stability of NDP-containing polymers. As Zhang and co-workers reported, two NDP-containing polymers **P50** and **P51**, both of which have narrow band gaps and suitable FMO energy levels, showed electron-dominated ambipolar transport behaviors, with the max electron mobility of 0.667 cm^2^ V^−1^ s^−1^ achieved by **P50**.^69^ The relatively better performance of **P50** was attributed to the lack of side-chain-induced backbone nonplanarity, which affected the chain orderly packing and the formation of large crystalline domains. Though ambipolar NDP polymers were obtained, unipolar n-type semiconductor materials based on the NDP unit were still unreported until the NDP-based polymer **P52** was synthesized, whose NDP block had no alkyl sidechain substitutions.^70^ In comparison with its sidechain-substituted counter polymer, **P52** showed more apparently bathochromic-shifted UV-vis absorption spectra and narrower bandgaps due to the enhanced intramolecular interaction between imide N and O atoms. Therefore, the **P52** film afforded unipolar n-type transport performance, with the mobility 40 times higher than its counter polymer.

In addition to modifying the conjugation length of the DPP core and connecting two functional groups of the DPP unit with an aromatic ring, the double bond was creatively introduced to connect two lactam structures. The bipyrrolylidene-2,2′(1*H*,1′*H*)-dione (BPD) unit was firstly reported by Cai and co-workers, who achieved a hole mobility of 1.4 cm^2^ V^−1^ s^−1^ for BDP-based small molecules.^71^ Later, two thiophene-BPD-based polymers, **P53** and **P54**, were also reported.^72^ Benefitting from the good planarity and delocalized FMO orbitals, two polymers showed appropriate HOMO/LUMO energy levels and narrow band gaps. Despite the comparable energy levels, **P53** afforded ambipolar transport performance with hole/electron mobilities of 1.24/0.82 cm^2^ V^−1^ s^−1^, respectively, whereas **P54** afforded a unipolar p-type transport performance with a hole mobility of 1.37 cm^2^ V^−1^ s^−1^. Such different transport types of **P53** and **P54** were attributed to their different sidechain orientations, in which bimodal and edge-on orientations were adopted by **P53** and **P54**, respectively. In the last five years, some other studies have focused on BPD units, including siloxane-terminated side chain substitution,^73^ and copolymerization with varied donor units.^74^ Most of these polymers showed balanced ambipolar transport behaviors for their applications in FET due to the appropriate FMO energy levels. The apparently enhanced FET performance and the changed transfer type suggested the BPD framework a competitive candidate for promising building blocks to construct more applicable semiconductors. As the analog of BPD, the (*E*)-[4,4′-biimidazolylidene]-5,5′(1*H*,1′*H*)-dione (BID) unit with additional nitrogen atoms embedded in the lactam ring was also used as the building block, which induced stronger intermolecular interactions of the corresponding polymer mainchains, thereby achieving p-channel carrier transport behaviors for its corresponding polymers.^75^ Despite the moderate mobilities of those BID-based polymers, the effectively adjusted band gap and near-infrared absorption region could be helpful for OSC application, which still worthy of investigation in the future.

**Fig. 6:**
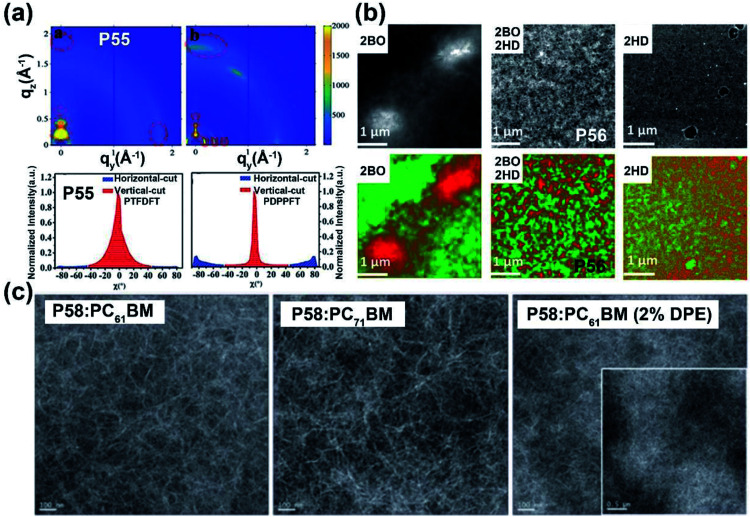
(a) 2D-GIWAXS patterns of **P55** and reference polymer thin films (up) and the corresponding pole figures (bottom). Reproduced from ref. 76 with permission from Wiley-VCH, copyright 2020. (b) Dark field STEM images (up) of the active layers containing **P56** and other two reference polymers, respectively, and the EELS maps of phase-separation patterns (bottom) between the electron-donor and electron-acceptor materials. Reproduced from ref. 79 with permission from Wiley-VCH, copyright 2018. (c) TEM images of different **P58**: PCBM thin films. Reproduced from ref. 81 with permission from the Royal Society of Chemistry, copyright 2019.

Most of the commonly investigated DPP structures had both their lactam nitrogen substituted by alkyl groups or other functional sidechains. Recently, **P55** was reported based on a novel building block, half-fused DPP, with one of the lactam nitrogen atoms embedded in a six-membered ring fused with the adjacent thiophene flanking unit.^76^ Besides the much more red-shifted absorption relative to the DPP-counterpart, the more rigid backbone and decreased steric hindrance provided the **P55** film with more ordered lamellar packing and denser π–π stacking, with the distances of 28.5 and 3.45 Å, respectively (Fig. 6a). The preferable polymer chain packing and the edge-on dominant bimodal orientation led to the max hole and electron mobilities of 1.08 and 2.23 cm^2^ V^−1^ s^−1^, respectively (Table 1). Efforts were also made to obtain DPP having both lactams fused to the adjacent units, with the promising solubility of alkylated thiophene flanking rings.^77^ Similar optical and chemical properties to the half-fused DPP were observed, which implied various applications in optoelectronic devices.

### 2.2. Polymers with IID and its derivatives

In the previous part, we comprehensively introduced semiconductor polymers based on DPP and its derivatives. As the other most investigated lactam-containing building block of polymers applied in optoelectronic devices, the IID unit was originally used in the medical and pharmaceutical fields. Thereafter, a series of six IID-based polymers was firstly synthesized by Stalder and co-workers. The systematic investigation of their physicochemical properties demonstrated the wide application of the IID unit.^78^ From then on, the IID unit has been developed to numerous analogs and derivatives, which could be generally divided into two main types. One type is monomers with a modified IID core. The other one is monomers with various conjugated bridges between their indolinone units.

#### 2.2.1. Polymers with modified indolinone units of IID cores

Since the IID unit was increasingly applied in optoelectronic devices, polymers with IID monomers had drawn much attention, and researchers had been devoted to optimizing its molecular structure to promote the performance in recent years. IID-based polymer **P56** had two IID units, which were substituted with 2-butyloctyl (2BO) and 2-hexyldecyl (2HD), respectively, and the molar ratio of 2BO : 2HD was 2 : 1.^79^ The **P56**:PBFTAZ blend film exhibited a max PCE value of 7.3%, much higher than those of 2BO-IID- and 2HD-IID-based polymers (Table 1). This was caused by the high degree of donor and acceptor mixing, which had evenly distributed phase-separation as revealed by electron energy-loss spectroscopy (EELS) (Fig. 6b). The alkylation was not only used to modify the electron-deficient unit. As shown in the **P57** structure, donor alkylation was also used to prepare polymers with its PCE value reaching 6.30% due to the improved film morphology.^80^ The further improvement of the PCE value of up to 10.7% was achieved by the polymer **P58**, which had both the sidechains of IID and thiophene ring elongated.^81^ Longer sidechains provided **P58** with good solubility in halogenated solvents and thereby facilitated the formation of bi-continuous phase separation and finer nano-fibrils in the film (Fig. 6c), implying the good contact for **P58** and PCBM. Likewise, synthesizing the more electron-deficient IID unit could have a significant influence on the device performance similarly to the alkylated IID units. The fluorine atom was incorporated into polymers **P59** and **P60**, both of which showed ambipolar transport behaviors.^82^**P59** exhibited the highest hole/electron mobilities of 3.49/2.77 cm^2^ V^−1^ s^−1^. The polymer **P60** gave an improved and more balanced ambipolar transport behavior with the max hole/electron mobilities of 3.94/3.50 cm^2^ V^−1^ s^−1^, respectively. Such enhancement could be associated with its more planar backbone, which was attributed to the more fluorine-related intramolecular interactions, such as F⋯H and F⋯S interactions, in addition, **P60** with a farther sidechain branching point exhibited an n-channel transport behavior with the max electron mobility of 4.97 cm^2^ V^−1^ s^−1^, which was caused by the decreased *d*-spacing and π–π stacking distance provided by multiple fluorinations and effective mainchain packing. To investigate the effect of different fluorination degrees on the polymers, **P61-P63** were constructed with the units IID, 1FIID, and 2FIID substituted with 0, 1, and 2 fluorine atoms, respectively.^83^ The optical band gaps, FMO energy levels, and their π–π distances decreased in the order of **P61**, **P62**, and **P63**. Such regularly changed properties led to their different transport mobilities. **P61** and **P63** showed hole-dominated and electron-dominated ambipolar transport behaviors, with the *μ*_h_/*μ*_e_ of 5.33/2.06 and 2.75/9.70 cm^2^ V^−1^ s^−1^, respectively. However, **P62** showed a more balanced transport performance with high *μ*_h_/*μ*_e_ values of 6.41/6.76 cm^2^ V^−1^ s^−1^. Their different FET performances indicated the adjustable transport types by varied backbone fluorination. Besides introducing fluorine atoms, nitrogen-embedded IID monomers also captured a lot of attention. As Huang and co-workers reported, the 7,7′-diazaisoindigo unit, in which 7-position carbon atoms were replaced by nitrogen atoms, possessed preferable backbone planarity.^84^ Besides, the corresponding polymer **P64** showed small torsional angles between its donor and acceptor moieties due to the S⋯N intramolecular interaction. Such a planar polymer backbone of **P64** agreed with its high hole mobility of 7.28 cm^2^ V^−1^ s^−1^.

**Fig. 7:**
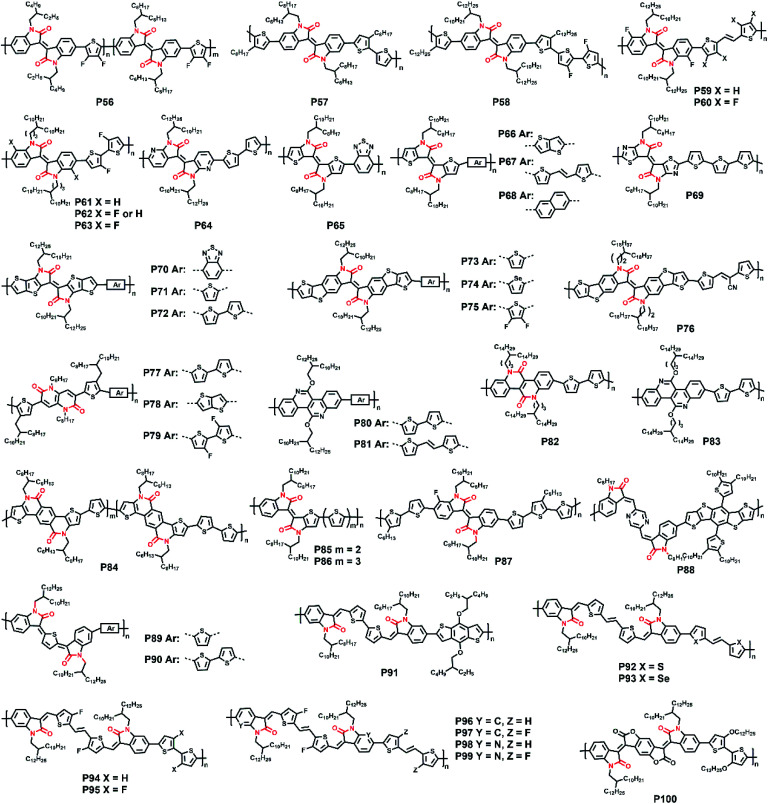
The molecular structures of polymers **P56–P100**.

**Fig. 8:**
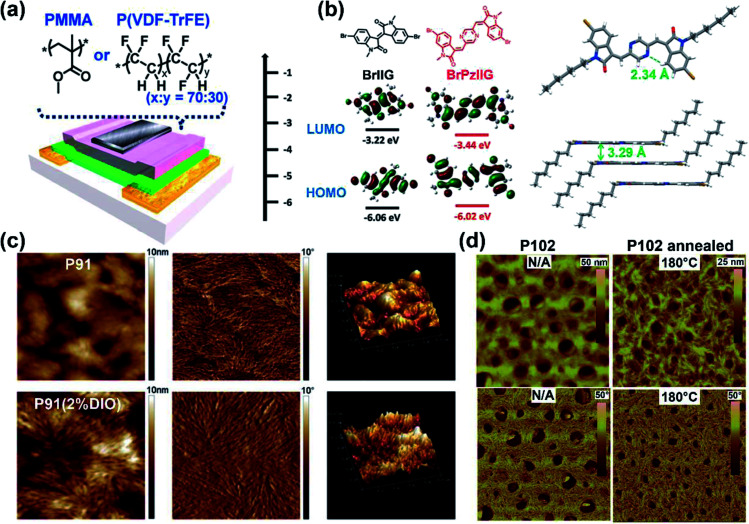
(a) The OFET device with the top-gate bottom-contact (TG-BC) configuration and the chemical structures of PMMA and P(VDF–TrFE) as the gate dielectric layer. Reproduced from ref. 87 with permission from the American Chemical Society, copyright 2014. (b) DFT estimated HOMO and LUMO energy levels of PzIIG and its counter monomer IIG (left) ant the single-crystal structures of the monomer PzIIG (right). Reproduced from ref. 100 with permission from the Royal Society of Chemistry, copyright 2017. (c) AFM images of the **P91**:PC_61_BM blend film (height images: left; phase images: middle) and the topography images (right). Reproduced from ref. 102 with permission from the Royal Society of Chemistry, copyright 2014. (d) AFM images of **P102** thin films before and after thermal treatment: height images (up), phase images (bottom). Reproduced from ref. 107 with permission from the Royal Society of Chemistry, copyright 2015.

The above electron-withdrawing atom substitution can effectively adjust the energy levels and relieve the backbone distortion of polymers. Apart from this, the aromatic rings fused with the lactam structure are also changeable for adjustment. Ashraf and co-workers replaced the benzene rings with thiophene rings in polymer **P65**, which afforded a balanced ambipolar transport behavior with mobility of around 0.1 cm^2^ V^−1^ s^−1^.^85^ Thereafter, **P66** and **P67** were synthesized based on thienoisoindigo units, both polymers showed improved mobilities of 0.39 and 0.45 cm^2^ V^−1^ s^−1^, respectively, compared with that of **P65**, due to their enhanced film crystallinity.^86^ As another thienoisoindigo-based polymer, **P68** exhibited an impressive charge carrier mobility.^87^ Different from the thiophene-contained donors in **P66** and **P67**, the naphthalene donor of **P68** facilitated the well-ordered layer-by-layer lamellar packing state with both edge-on and face-on crystal domains, thus achieving a high *μ*_h_ up to 14.4 cm^2^ V^−1^ s^−1^ in the device with the high-dielectric-constant poly(vinylidenefluoride-trifluoroethylene) as the gate dielectric, which promoted accumulated positive charge carriers induced by the multiple fluorine atoms of the new dielectric (Fig. 8a). Similarly, further N-embedding modification of the thienoisoindigo core was completed by Li and co-workers, who synthesized the polymer **P69**.^88^ Owing to the smooth surface of the dominant edge-on orientation content of the film, the **P69** film gave the max *μ*_h_/*μ*_e_ of 3.93/1.07 cm^2^ V^−1^ s^−1^ in its TGBC FET devices, and unipolar p-type transport performance with mobility of 3.41 cm^2^ V^−1^ s^−1^ was also achieved in BGTC devices. As shown in Fig. 7, the polymers **P70-P72** contained the extended thienoisoindigo, thieno[3,2-*b*] thiophene isoindigo (IIDTT), as the acceptor unit.^89^ The extended backbone facilitated an increased backbone coplanarity, which resulted in the more effective π-orbital overlap. Therefore, **P70-P72** exhibited ambipolar transport performances and **P70** showed the highest *μ*_h_/*μ*_e_ of 0.4/0.7 cm^2^ V^−1^ s^−1^, respectively. Yue and co-workers further fused IIDTT with additional thiophene rings and synthesized a novel building block, thieno[3,2-*b*][1]benzothiophene isoindigo (TBTI).^90^ The corresponding polymer **P73** showed strong XRD diffraction peak intensity with a large coherence length, demonstrating that the further extension of the conjugated length effectively improved the film order and the crystallinity. Hence, both the PCE of 9.1% and the hole mobility of 0.31 cm^2^ V^−1^ s^−1^ were achieved in its OSC and OFET devices, respectively. The TBTI-based polymers **P74-P76** were also synthesized with high PCE values surpassing 5.0%, which could be attributed to the well-distributed small domain size of **P74-P76** and the PC_70_BM in their respective blend films and their appropriate ionization potentials and electron affinities.^91^

Besides the five-membered lactam structure, the six-membered bis-lactam monomer 1,4-naphthyridine-2,6-dione (NTD) was used as the acceptor in **P77-P79**.^92^ The intramolecular S⋯O interaction improved the molecular coplanarity and all three polymer films adopted face-on orientation with small π–π stacking distances. **P79**, with the smallest π-stacking distance of 3.61 Å, exhibited the highest PCE up to 9.63%. Owing to its stronger electron-accepting capacity and structural planarity, such a six-membered lactam moiety was widely modified and used. **P80** and **P81** featured novel acceptor dibenzo[*c*,*h*][2,6]naphthyridine-5,11(6*H*,12*H*)-dione (DBND), which had further benzene rings fused with the NTD unit.^93^ The only difference was that the DBND unit adopted O-alkylation rather than common *N*-alkylation. Despite the large dihedral angles of 30° existing between DBND and donor moieties, both **P80** and **P81** had short π–π distances of 3.74 and 3.38 Å, respectively, and the denser film packing gave a higher PCE value of 6.32% for **P81** than the 5.75% of **P80**. Subsequently, they disclosed the other two DBND-based polymers **P82** and **P83** applied in FET transistors.^94^ The *N*-alkylated **P82** exhibited a more red-shifted UV-vis absorption spectrum and had a larger *I*_0−0_/*I*_0−1_ value compared with the *O*-alkylated **P83**, implying a tighter aggregation of **P82**. Moreover, **P82** had higher intermolecular self-assembly binding energy due to its stronger intermolecular interaction. The above results led to the smaller π–π distance and interconnected crystalline zones in the **P82** film, which was consistent with its better unipolar p-type transport performance with a hole mobility of 0.55 cm^2^ V^−1^ s^−1^. Furthermore, **P84** used thieno[2′,3′:5′,6′]pyrido[3,4-*g*]thieno[3,2-*c*]isoquinoline-5,11(4*H*,10*H*)-dione (TPTI) as the acceptor unit due to its strong electron-accepting ability and structural planarity.^95^ The terpolymer **P84**, with 70% TPTI-thiophene, afforded the highest PCE up to 11.02%, which was about 20% higher than those of other polymers with different ratios of donors. In comparison with all these blend polymer films (polymer: *m*-ITIC) having bimodal backbone orientation, the **P84**-based blend film exhibited more percentage of face-on orientation, which had vertical charge-transportation channels for better photovoltaic application.^96^

Just as the asymmetrically constructed DPP units, the IID unit and its derivatives could also have their indolinone moieties changed. To relieve the steric hindrance in the twisted indolinone moieties around the C=C bond of the isoindigo monomer, **P85** and **P86** were developed on thieno-benzo-isoindigo (TBIID).^97^ The optical bandgaps and FMO energy levels of **P85** and **P86** fell between those of IID- and thieno-IID-based counter polymers. The decreased torsional angles between two indolinones were around 0.11 and 0.19° for **P85** and **P86**, respectively, favoring well-ordered polymer packing and the formation of large crystalline domains. Both **P85** and **P86** exhibited unipolar p-type transport properties and **P86** afforded a max PCE of 4.79% in its OSC devices. The TBIID unit was still widely used in polymers nowadays for its good OSC performance guarantee.^98^ Besides the asymmetrical fused rings for isoindigo, there was also some other way of asymmetrical combination. The asymmetrical fluorine substitution was carried out in **P87**, in which one of the indolinone units had fluorine substitution on its 7th carbon atom.^99^ The **P87**:PCBM film gave a PCE value of 5.03%, slightly higher than those of the above-mentioned TBIID-based polymers (Table 1). This is because the fluorine-related inter- and intra-chain interactions could not only facilitate better backbone planarity but also form a well-mixed film with PCBM.

#### 2.2.2. Polymers based on IID derivatives with conjugated bridges

In the above part, the modification of the IID unit is mainly performed on its indolinone units. Nevertheless, the replacement of the phenyl ring with thiophene or the asymmetric structure strategy of the TBIID monomer could partly relieve the steric repulsion between the hydrogen of benzene and the oxygen of the carbonyl group. Additionally, connecting two indolinones with aromatic rings rather than the carbon–carbon double bond has been extensively studied to not just reduce the steric hindrance but to construct semiconducting polymers with extended π-conjugation, hence achieving favorable properties.

The polymer **P88** contained an acceptor PzIIG, having the 1,4-pyrazine ring as the bridge between two indolinones.^100^ The PzIIG had little difference in the HOMO/LUMO energy levels relative to those of the IID counterpart but gave a dense and ordered packing state, whose π–π stacking distance was as small as 3.29 Å (Fig. 8b), due to the formed N⋯H hydrogen bond between 1,4-pyrazine and indolinone. The **P88**:PC_61_BM blend film exhibited a max PCE of 3.12%, which was further enhanced to 5.26% after adding 3% additive of 1,8-diiodooctane and changing PC_61_BM to PC_71_BM (Table 1). Similarly, quinoidal polymers **P89** and **P90** were obtained, with their IID indolinone units connected with a thiophene ring.^101^ The extended quinoidal acceptor provided the two polymers with narrow optical band gaps. Though there were torsional angles of around 26° between inserted thiophene and indolinone units for both **P89** and **P90**, the annealing treatment facilitated two polymer films to adopt favorable edge-on orientations with strong π–π interactions. Therefore, their off-center spin-coating films afforded the max hole/electron mobilities of 4.82/1.11 cm^2^ V^−1^ s^−1^ for **P89** and 8.09/0.74 cm^2^ V^−1^ s^−1^ for **P90**, respectively. Such outstanding performances could be attributed to the centrifugal force, which led to orderly aggregated polymer chains and improved crystallinities.

**Fig. 9:**
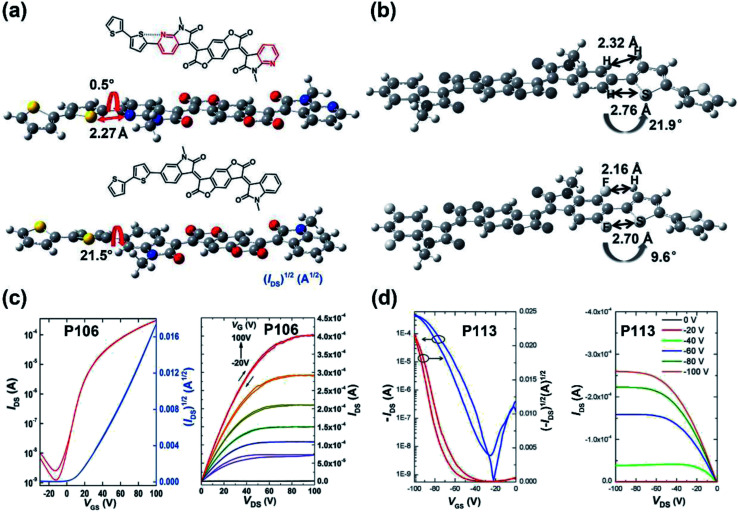
DFT-optimized structures with marked dihedral angles and the intramolecular interaction distances of the AzaBDOPV-2T fragment (a, up), Reproduced from ref. 108 with permission from the Royal Society of Chemistry, copyright 2016, Jian Pei, *et al.*, licensed under CC BY 3.0, https://creativecommons.org/licenses/by/3.0/. BDOPV-2T fragment (a, bottom/b, up), and F_4_BDOPV-2T fragment (b, bottom). Reproduced from ref. 110 with permission from Wiley-VCH, copyright 2016. (c) The transfer (left) and output (right) characteristics of **P106**-based FET devices. Reproduced from ref. 110 with permission from Wiley-VCH, copyright 2016. (d) The transfer (left) and output (right) characteristics of **P113**-based FET devices. Reproduced from ref. 115 with permission from the Royal Society of Chemistry, copyright 2015.

Likewise, the acceptor IBTI, with two indolinones connected with a bithiophene unit, was used in **P91** × ^102^ The S⋯N intramolecular interactions relieved the molecular distortion and provided a well-delocalized HOMO over the entire backbone. It was found that the highest PCE value of 3.49% was achieved when the weight ratio of **P91** : PC_61_BM reached 1 : 2. With additional DIO, the **P91**:PC_61_BM blend film showed well-proportioned and interpenetrated morphology with good miscibility (Fig. 8c) and finally gave a considerably improved PCE of 6.41%. Considering the rotatable single bonds in bithiophene units, the vinyl groups were introduced into bithiophene to form a stable planar backbone in the obtained VDTOI units and two polymers **P92** and **P93**.^103^ The increased planarity helped obtain smooth and uniform polymer films. Both **P92** and **P93** showed p-type transport performances and the highest hole mobility of 0.35 cm^2^ V^−1^ s^−1^ was achieved by the **P92** film, due to its higher crystallinity and effective molecular packing state with the π–π stacking distance of 3.71 Å. More recently, the fluorinated TVT moiety and nitrogen-embedded indolinone were combined to afford four polymers **P94-P97**.^104^ With much-lowered FMO energy levels and ignorable torsional angles provided by multiple intrachain interactions, all the polymer films showed p-type transport performances. **P97** and **P98** exhibited the highest *μ*_h_ of 0.48 and 0.47 cm^2^ V^−1^ s^−1^, respectively, attributed to their relatively smaller π-stacking distances of around 3.50 Å. Nonetheless, all those polymers featured unipolar p-type transport performance, whatever the conjugated bridge was bithiophene or TVT unit. This is because their electron-donating nature could hardly tune the LUMO energy level to a preferable one for efficient electron injection. To settle down such a problem, the benzo[1,2-*b*:4,5-*b*′]difuran-2,6(3*H*,7*H*)-dione unit was inserted between two indolinones, leading to a more electron-deficient building block (IBDT) in **P100**.^105^ The polymer **P100** had much red-shifted absorption spectra with the optical band gap approaching 0.95 eV. Such a narrow energy gap gave the **P100** film a highly balanced hole and electron mobility of 0.16 and 0.14 cm^2^ V^−1^ s^−1^, respectively. Higher mobilities were achieved by the other IBDT-based polymer **P101**.^106^ The more rigid thieno[3,2-*b*]thiophene donor contributed to the high crystallinity and ordered interchain packing of the **P101** film, which adopted edge-on orientation with the π–π stacking distance of 3.45 Å. All these results resulted in its high *μ*_h_/*μ*_e_ of 1.70/1.37 cm^2^ V^−1^ s^−1^, respectively. When the outer benzene rings in IBDT indolinone moieties were replaced by thiophene rings, the resulting monomer in **P102** featured a more planar backbone due to the S⋯O interaction.^107^ In comparison with its reference polymer and analog polymer **P100**, the thiophene replacement provided the **P102** with a lowered LUMO energy level and enhanced HOMO energy level, leading to its ultra-narrow band gap of 0.71 eV. Besides, the annealed **P102** film showed a large and continuous nano-fibrillar structure, with an interconnected network, rather than clustered crystals in its as-cast film (Fig. 8d). For the above reasons, **P102** afforded higher hole and electron mobilities of 0.45 and 0.22 cm^2^ V^−1^ s^−1^, respectively, relative to the mobilities of **P100**. It has been proved that azaisoindigo was preferable for improving the polymer backbone planarity and charge carrier transport performance.^108^ Likewise, nitrogen-embedded IBDT (AzaBDOPV)-based polymer **P104** was prepared, with a much lowered LUMO energy level and smaller dihedral angles than those of its IBDT-based counter polymer **P103** (Fig. 9a). The typical n-type transport performance was achieved for **P104**, with the highest mobility of 3.22 cm^2^ V^−1^ s^−1^, much higher than that of **P103**. Such better carrier transport performance was attributed to its shorter π–π distance of around 3.44 Å and its strong crystallinity with fiber-like crystallized zones. Later, when the AzaBDOPV and dithienylbenzothiadiazole units were copolymerized, the resulting polymer **P105** afforded balanced FET performance with greatly enhanced mobilities compared to that of **P104**.^109^ Using 1-octanethiol self-assembled monolayer modified source and drain electrodes, the spin-coated **P105** film displayed the highest hole/electron mobilities of 5.97/7.07 cm^2^ V^−1^ s^−1^, respectively, in its TGBC FET device under ambient conditions. Moreover, **P105**-based flexible FET devices gave balanced hole/electron mobilities of 4.68/4.72 cm^2^ V^−1^ s^−1^. The denser film stacking state and increased crystallinity of the **P105** film, with a short π–π stacking distance of 3.46 Å and clear grains with fiber-like polycrystalline networks, could be helpful to realize its favorable carrier mobilities. At present, incorporating multiple electron-withdrawing atoms in the polymer backbone was proved to be effective for achieving satisfactory optoelectronic properties. In polymer **P106**, F_4_BDOPV with multiple fluorine substitutions was chosen as the acceptor.^110^ The numerous fluorine atoms in **P106** formed both the F⋯H hydrogen bond and F⋯S intramolecular interaction, which led to less backbone distortion than its unfluorinated counter polymer (Fig. 9b), and synchronously increased the interchain packing with a small π–π stacking distance of 3.52 Å. The good crystallinity provided the **P106** film with an electron mobility of 1.24 cm^2^ V^−1^ s^−1^ (Fig. 9c), which could still maintain 60% after 30 days of exposure under ambient conditions. Such promising good air-stability could be illustrated by its deep-lying energy level.

Several modifications of indolinone units were developed, including the incorporation of electronegative atoms, the extension of conjugation, and the replacement of structural building blocks. Meanwhile, researchers endeavored to develop novel inserting core units like structural analogs to IBDF, possessing a planar backbone, extended π-system, and stronger electron with-drawing capacity. Then the novel building block IBDT, in which the benzodifurandione moiety of the IBDF unit was replaced with the benzodithiophenedione moiety, came into the sight of scientists. **P107**, as an IBDT-based polymer, had a comparable HOMO energy level to the IBDF-based counter polymer, but with a lower-lying LUMO energy level due to its smaller exciton binding energy.^111^ Finally, the **P107** film gave a highly balanced ambipolar carrier transport property with the highest *μ*_e_/*μ*_h_ of 0.14/0.10 cm^2^ V^−1^ s^−1^, respectively. To achieve ambipolar transport behavior as **P107** did but with higher mobilities, π-extended IBDF (INDF) was used in **P108** and **P109**.^112^ Compared with IBDF, the newly introduced naphthalene ring provided the INDF with an extended conjugation length and a weaker electron-accepting ability. Such changes favored stronger intermolecular interaction and relatively higher FMO energy levels hence achieving more balanced ambipolar FET performances. Though the dihedral angles between the indolinone and central core were slightly increased 3–5° relative to that of IBDF, smooth films with edge-on orientation motif were observed for both the polymers, thus exhibiting highly balanced ambipolar transport performances, with the max *μ*_e_/*μ*_h_ of 0.14/0.12 cm^2^ V^−1^ s^−1^ for **P108** and 0.50/0.51 cm^2^ V^−1^ s^−1^ for **P109**. Later, the different sidechains were substituted in both donor and acceptor units of another INDF-based polymer, **P110**.^113^ The increased solubility of the whole polymer backbone gave a much apparent red-shifted absorption compared to that of **P109**, indicating a stronger tendency of mainchain aggregation in solution and denser film packing in the solid state. Finally, good FET performance of **P110** film was obtained, with the max *μ*_e_/*μ*_h_ of 0.15 and 0.33 cm^2^ V^−1^ s^−1^, respectively, which were comparable to those of most polymers with INDF.

**Fig. 10:**
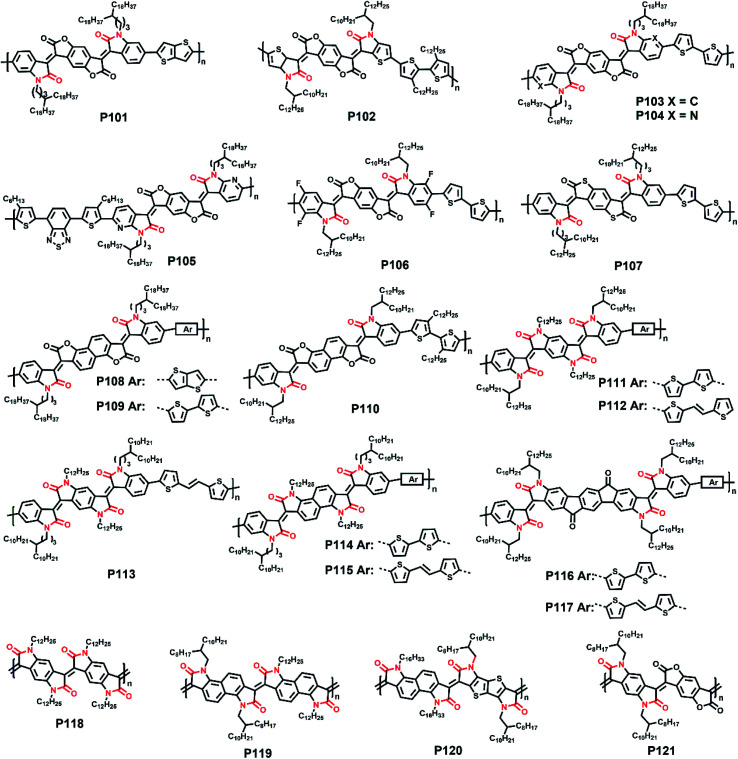
The molecular structures of polymers **P101–P121**.

The improved solubility was proved to be capable of enhancing polymer device performance by adjusting their molecular packing states, and therefore, more available alkylation positions can provide sufficient sidechain modification. For instance, **P111** and **P112** contained the lactam-modified building block IBDP.^114^ The two more lactam structures in the acceptor allowed the application of shorter substituted side chains to enhance solubility and the polymer solution processability, without sacrificing the coplanarity. The promising sidechain self-assembly enabled the fully stretched sidechain and produced long *d*-spacing distances, which reached 27.1 Å for the **P111** film and 25.5 Å for the **P112** film, respectively. Both **P111** and **P112** showed ambipolar transport performances with their respective *μ*_e_/*μ*_h_ of 0.088/0.19 and 0.075/0.10 cm^2^ V^−1^ s^−1^. The four lactam moieties of the IBDP monomer ensured its modifiable solubility and mainchain packing state by diverse sidechain substitutions. After moving the branching point of the sidechain in indolinone away from the backbone of **P113**, it showed a better lamellar edge-on packing state than **P111** and **P112** did, thereby facilitating stronger interchain packing.^115^**P113** eventually exhibited typical p-type transport performance and exhibited higher mobility up to 1.92 cm^2^ V^−1^ s^−1^ owing to the fiber-like networks of its film morphology (Fig. 9d). Just like the extension of IBDF to obtain the INDF monomer, the same strategies were applied in the polymers **P114** and **P115**, in which the phenyl-extended IBDP served as the electron-deficient building block.^116^ Such change had their HOMO energy levels raised to around −5.20 eV, which was preferable for hole carrier injection, thereby leading to their hole-dominated ambipolar performances with the max *μ*_h_/*μ*_e_ of 1.55/0.021 cm^2^ V^−1^ s^−1^ for **P114** and 1.79/0.087 cm^2^ V^−1^ s^−1^ for **P115**, respectively (Fig. 10).

Generally speaking, the above building blocks, including IBDF, INDF, and other analogs, could be mainly regarded as two types of connection between IID units. One is that two IID units were fused by sharing one benzene ring (*e.g.*, **P111-P113**) and the other one was the direct fusion of two IID units (*e.g.*, **P114-P115**). Moreover, the third type of such kind of acceptor was incorporated in the polymers **P116** and **P117**, in which two IID units were fused with the 2,3,6,7-tetrahydro-*s*-indacene-1,5-dione moiety by intramolecular Friedel–Crafts acylation of carboxylic acids.^117^ The extended backbone and additional carbonyl groups facilitated the delocalization of both the HOMO/LUMO orbitals and lower energy levels. **P116** and **P117** showed balanced ambipolar transport performances with both the highest hole and electron mobilities of around 0.10 cm^2^ V^−1^ s^−1^, achieved by **P117** due to its more percentage of edge-on orientation crystal domains.

Owing to the current polymerization mechanism, like Stille, Suzuki–Miyaura, and Kumada coupling, the traditional bonding way between aromatic rings is always associated with the single bond, which potentially leads to the backbone distortion and causes the energetic disorder.^118^ To settle down such a problem, Onwubiko and co-workers utilized a new synthetic approach and disclosed a series of polymers, **P118-P120**.^119^ The way of aldol polymerization enabled the building blocks to connect *via* the double bond. The resulting rigid backbone and planar backbone resulted in apparently broadened absorption spectra and more red-shifted absorption peaks in the region from 850 to 1200 nm. All the polymers exhibited unipolar n-type transport performances and the highest air-stable electron mobility of 0.03 cm^2^ V^−1^ s^−1^ was achieved by **P119**. Later, another ladder polymer **P121** was also reported, whose two building blocks were combined only with a double bond.^120^ Simultaneously, good backbone planarity was achieved due to the multiple double bonds and the intramolecular hydrogen bonds between two adjacent units. The **P121** film eventually displayed unipolar n-type transport performance with the highest electron mobility of 0.18 cm^2^ V^−1^ s^−1^ (Table 1). Though all those polymers did not show good performance in FET devices as expected, which could be possibly attributed to the unideal sidechain self-assembly in their films caused by the rigid-backbone-induced quick interchain packing process, such a double-bond strategy can be used in those block copolymers for adjustment in the film morphology and the device performance.

## 3. Polymers with imide moieties

Besides DPP and IID monomers, there are some other novel electron-deficient building blocks that featured imide functional groups. Compared with the lactam moiety, additional carbonyl groups of the imide moiety make the molecule more electron-deficient. Hence naphthalene diimide (NDI), perylene diimide (PDI), and some other imide-functionalized acceptors were increasingly used to construct polymer semiconducting materials. Though studies on imide-functionalized polymers were not as early as their corresponding small molecules, their photoelectric performances were rapidly improved and exceeded those of small molecules as their chemical structures were continuously modified.

### 3.1. Polymers based on NDI and its derivatives

Featuring multiple aromatic ring fusion and dual electron-withdrawing imide functional groups, the NDI unit is the ideal electron-deficient building block for semiconducting polymer materials and has been widely investigated. Especially in recent several years, many corresponding analogs and derivatives have been designed and reported to fabricate optoelectronic-device-favorable polymers.

The first NDI-based polymer, reported by Guo and co-workers,^121^ showed promising physical and chemical properties, which attracted the interest of scientists around the world. Later, Yan and co-workers synthesized the polymer **P122**, which afforded the electron mobility up to 0.85 cm^2^ V^−1^ s^−1^ under ambient conditions (Table 1).^122^ Thereafter, wide applications in organic solar cells made it well known as N2200. Until today, N2200 is still the most common material used for fabricating high-performance OSC devices. More recently, N2200-containing all-polymer solar cells (all-PSCs) successfully afforded a considerably impressive PCE value of over 10% on 1 cm^2^ device area.^123^ Owing to the excellent properties and various applications of N2200, there were numerous attempts to synthesize a better polymer through structural modification. One of the successful attempts was F-N2200, which was obtained by substituting two fluorine atoms onto the bithiophene unit of N2200.^124^ Owing to the strengthened intramolecular stacking and more effective Si-polymer contact provided by the additional F–H interaction, Si-PEDOT:PSS-based solar cells with F-N2200 as the interlayer showed higher PCE up to 14.5% than 12.6% of its N2200 counterpart. When additional well-soluble donors were introduced, **P123** and **P124** were obtained.^125^ Benefitting from their amine or ammonium bromide group substitution, both the polymers showed good solubility in water and alcohol, which enable different films to be obtained with varied thicknesses. 5 nm-thick **P123** and **P124** films eventually exhibited the best OSC performances with the max PCE of 9.42% and 10.11%, respectively. In fact, shortly after the report of the first NDI-based polymers, a high electron transport mobility of up to 6.0 cm^2^ V^−1^ s^−1^ was achieved by the NDI-based small molecule.^126^ Nevertheless, the NDI-based polymer with high FET mobilities was not reported until 2017, when Zhao and co-workers reported four NDI-based conjugated polymers, **P125-P128**.^127^ All polymers had broad UV-vis absorption bands, indicating an interactive polymer backbone, and **P125**/**P126** showed broader ones due to the strong electronic coupling effect of selenophene. All polymer films adopted edge-on-dominated bimodal orientations with small π–π stacking distances (Fig. 12a). **P125** and **P127** exhibited ambipolar transport properties with the highest *μ*_e_/*μ*_h_ of 8.5/1.7 and 3.1/0.7 cm^2^ V^−1^ s^−1^, respectively, whereas their fluorinated counter polymers **P126** and **P128** afforded unipolar n-type transport performances with the max *μ*_e_ of 3.5 and 2.2 cm^2^ V^−1^ s^−1^, respectively. Such differences were caused by the increased hole injection barrier of fluorinated **P126** and **P128**, thereby curtailing the injection of the hole carrier and facilitating n-channel carrier transport performances. For well optimizing the backbone conformation, vinylene spacers were introduced into **P129** and **P130** to form multiple hydrogen bonds.^128^ The improved planarities resulted in short π–π stacking distances for **P129** and **P130**, thus facilitating electron-dominant ambipolar transport behaviors, with the respective high electron mobilities of 3.95 and 7.37 cm^2^ V^−1^ s^−1^ being obtained from the transfer and output characteristics of **P129** and **P130** (Fig. 12b). The much higher electron mobility of the **P130** film could be explained by its dense π–π stacking in both out-of-plane and in-plane two directions with the small distances of 3.45 and 3.40 Å (Fig. 12c).

The NDI core was constituted by several fused rings. Further heteroaromatic-fusion on it achieved a planar and rigid backbone for good interchain π–π stacking. The thiophene-fused NDI (NDTI) unit in **P131** showed a highly planar backbone, having a one-dimensional columnar structure with the π–π stacking distance of around 3.43 Å in its single-crystal structure.^129^ This preferable packing state provided the **P131** film with ambipolar performance with *μ*_h_ and *μ*_e_ of 0.10 and 0.27 cm^2^ V^−1^ s^−1^, respectively, indicating the comparable application potential of the NDTI unit to the NDI unit. Moreover, **P132**, in which NDTI was combined with dithienothiophene, featured a much red-shifted absorption and a PCE of 5.57%, which was over twice as high as that of the NDI-based counter polymer.^130^ Using the same NDTI unit in the synthesis of the polymer **P133-P135**, Wang and Takimiya incorporated bithiopheneimide and thiazole units to achieve different polymer backbone geometries.^131^ Different from the wave-line backbone of **P133**, thiazole incorporation changed the backbones of **P134-P135** to a straight-line geometry (Fig. 13a), which induced improved intermolecular packing (Fig. 13b) and hence the FET performance. Despite the ambipolar transport behaviors of all the polymers, **P134** and **P135** had much-improved electron mobilities. **P135** afforded an electron mobility of 0.55 cm^2^ V^−1^ s^−1^, which was nearly a hundred-fold higher than that of **P134** and also suppressed most of the other NDTI-containing polymers. Such results for **P134-P135** were also associated with their different backbone orientations, in which the farther branching point provided **P135** with bimodal orientation rather than the face-on and edge-on orientation of **P133** and **P134**, respectively (Fig. 13c).

The extended NDI backbone contributed to a more extended conjugation and low-lying LUMO energy level of polymers, which were preferable for FET application. However, low LUMO levels of NDI-based polymers led to low *V*_oc_ (<0.6 V) for OSC devices. Therefore, the 1,2,5,6-naphthalenediimide (1,2,5,6-NDI) unit, with less aromatic fused rings, was built up in **P136** and **P137**.^132^ The decreased backbone electron deficiency enabled both polymers to have raised LUMO energy levels of around −3.30 eV, which successfully enhanced their *V*_oc_ to above 0.9 V, with the highest PCEs of 5.04% for **P136** and 6.35% for **P137**. These results showed a big influence of the different unit extensions on the final performance of their corresponding polymers. Besides, the controllable copolymerization site was also proved to be efficacious, *e.g.*, **P138** and **P139** based on the novel electron-deficient building block dithieno[3,2-*a*:3′,2′-*j*][5,6,11,12]chrysene diimides (DTCDI) had more extended π-conjugation compared with 1,2,5,6-NDI.^133^ The electron-deficient nature of DTCDI resulted in much lower LUMO energy levels of **P138** and **P139**, and more importantly, its unique structure ensured different reaction sites. The DTCDI-3,9-connected **P138** film displayed uniform fibrous nanostructures with a larger grain size than that of the DTCDI-thiophene α,α′-connected **P139** film. Such differences explained their huge different device performances. **P138** showed a PCE of 7.52% in its OSCs and a FET electron mobility reaching 0.11 cm^2^ V^−1^ s^−1^, which was greatly higher than that of **P138**. The dual imide structures in the above-mentioned electron-deficient building blocks were symmetrically constructed in the skeleton, thus facilitating even distribution of FMO orbitals. With only one imide moiety located at one side of the main chain, the polymer **P140** was developed based on a single-imide-featured TCNDIO unit.^134^ Despite one less imide structure of TCNDIO, **P140** also had a wide absorption band range with a narrow optical band gap of 1.06 eV and comparable energy levels to other dual-imides-functionalized polymers. Because of a small torsional angle of 10.6° in the TCNDIO monomer, the **P140** film adopted face-on orientation with the π–π stacking distance of around 3.79 Å. Finally, **P140** showed a p-channel charge transport behavior with a hole mobility of 0.02 cm^2^ V^−1^ s^−1^. From another perspective, such a strategy could be further optimized to achieve a decreased density of side-chain substitution thus facilitating more effective intermolecular mainchain stacking.

Different from the original extension position of the NDI unit, in which the aromatic rings were fused next to the phenyl, the thienopyridine-fused naphthalene amide (TPNA) unit was obtained by fusing at the diagonal position of the NDI unit.^135^ Three types of polymers (homopolymer, D–A polymer, and all-acceptor polymer) were synthesized, namely **P141**, **P142**, and **P143**, with deep-lying HOMO energy levels ranging from −5.99 to −5.76 eV. The promising backbone planarity and π-conjugation conferred unipolar electron transport properties to all three polymers. Compared with **P141** and **P142**, **P143** had better planarity, lower-lying HOMO energy levels, and a closer π–π stacking distance of 3.49 Å, hence exhibiting better FET performance with the highest *μ*_e_ of 0.19 cm^2^ V^−1^ s^−1^ (Table 1).

Obviously, NDI-based polymers have both advantages and disadvantages. The brominated NDI monomer has less synthetic complexity, high yield, and a simple purification process. These positive aspects promise convenient access to varieties of polymers containing NDI or its structural analogs. NDI-based polymers feature good air-stable electron transport behaviors due to their deep-lying LUMO energy levels. Although NDI and its derivatives are widely applicable, there are still some drawbacks in them. The molecular structures of NDIs curtail the good coplanarity of the polymer main chain backbone, which makes most NDI-containing polymers have their LUMO located only at the NDI units, thus leading to much lower charge carrier mobilities than those of DPP- or IID-based counter polymers. And such drawbacks might be the main thing to surmount in the nearest future.

### 3.2. Polymers based on PDI and its derivatives

**Fig. 11:**
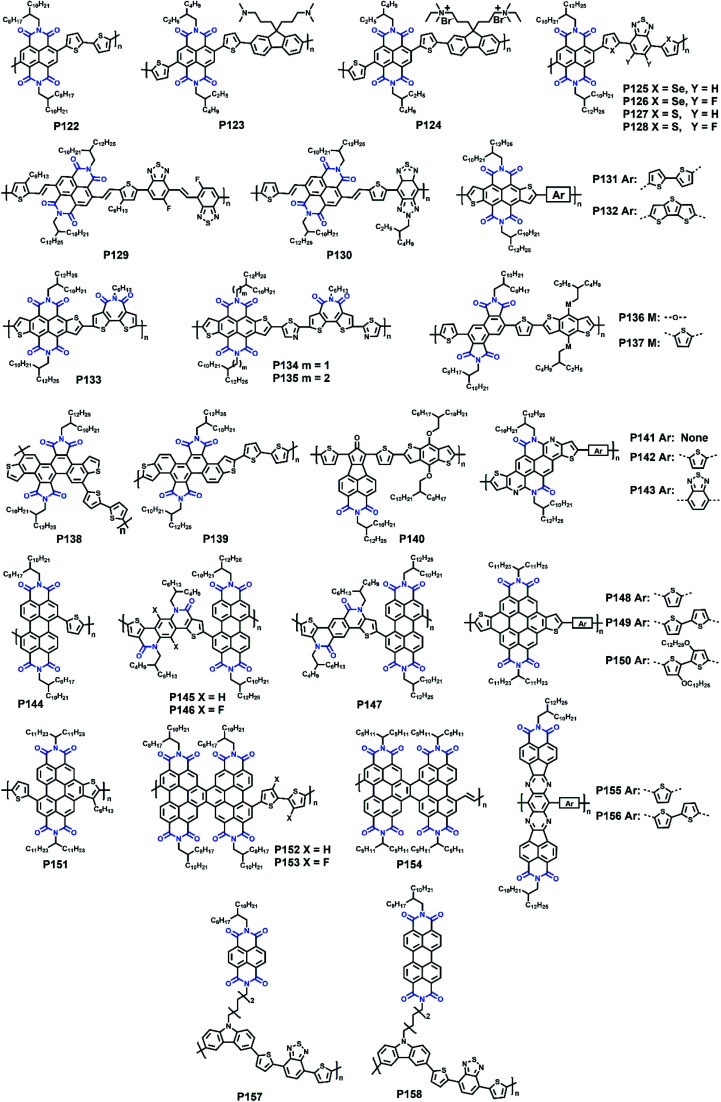
The molecular structures of polymers **P122–P158**.

The PDI unit is also another popular imide-functionalized electron-deficient building block for application in semiconductor polymers. Compared with the NDI unit, the PDI unit is structurally similar but has more fused benzene rings and has been investigated earlier. In 2007, Zhan and co-workers firstly reported a PDI-based polymer, exhibiting good solubility and thermal stability.^136^ Such polymers afforded n-type FET performance (1.3 × 10^−2^ cm^2^ V^−1^ s^−1^) and also gave a max PCE over 1% in its OSC devices, both of which were at high levels at that time. Since then, numerous semiconducting polymers have been developed based on PDI and its derivatives, especially in recent several years. As shown in Fig. 11, polymer **P144** showed good solubility in green solvents due to its long side chain in the NDI unit.^137^ The much-increased solubility enabled the anisole-processed PBDT-TS1:**P144** film to afford a max PCE of 5.43% and an improved PCE of 6.58% in its inverted all-PSCs (Fig. 14a and Table 1), both of which were comparable to the highest value achieved by the film treated with halogenated solvent. Polymers with various D–A pairs, A–A type polymers, **P145-P147**, were also reported based on three available stannylated units (TPTQ, FTPTQ, and TPTI), containing lactam moieties.^138^ In comparison with the FMO energy levels of other PDI-based polymers, the additional acceptor units in **P145-P147** showed limited adjustment to their LUMO energy levels but greatly dragged down their HOMO energy levels. The different internal polarization resulted in their different PCE values and **P145**-based devices afforded the highest PCE of 3.52% among the three polymers, which could be explained by its stronger face-on orientation after modification and the better binding affinity with the electron-donating material PTB7-Th.

**Fig. 12:**
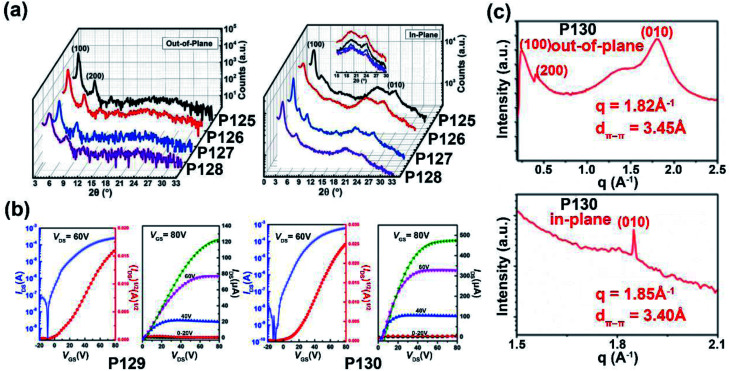
(a) The GIXRD profiles of **P125-P128** films (annealed at 200 °C) in the out-of-plane (left) and in-plane direction (right). Reproduced from ref. 127 with permission from Wiley-VCH, copyright 2017. (b) The transfer and output characteristics of **P129**- and **P130**-based OFET devices. Reproduced from ref. 128 with permission from the American Chemical Society, copyright 2019. (c) 1D profile of **P130** in the out-of-plane (up) direction and in the in-plane direction (bottom). Reproduced from ref. 128 with permission from the American Chemical Society, copyright 2019.

**Fig. 13:**
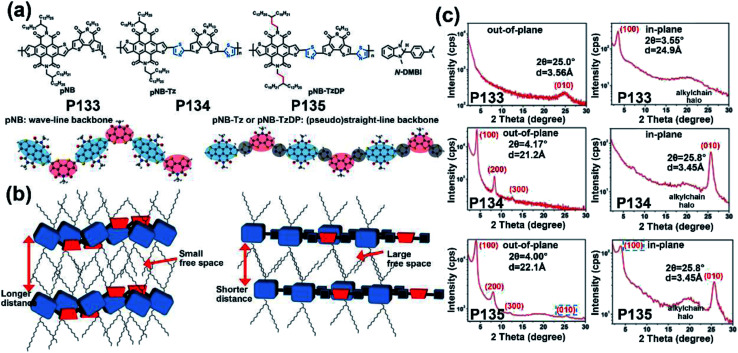
(a) Molecular structures of **P133-P135** and n-doped *N*-DMBI (up) and their optimized backbone geometries (bottom). Reproduced from ref. 131 with permission from Wiley-VCH, copyright 2020. (b) The illustration of interpolymer the docking models of **P133** (left) and **P134** (right). Reproduced from ref. 131 with permission from Wiley-VCH, copyright 2020. (c) 1D GIXRD patterns of **P133-P135** in the out-of-plane direction (left) and in the in-plane direction (right). Reproduced from ref. 131 with permission from Wiley-VCH, copyright 2020.

**Fig. 14:**
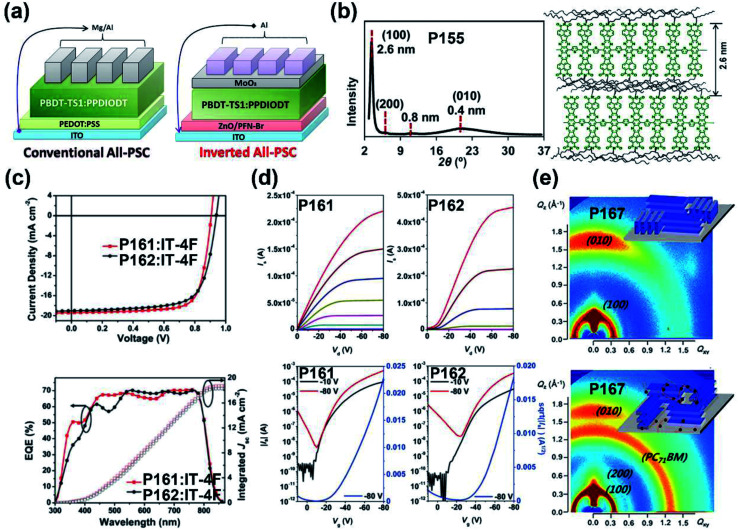
(a) The device architectures of the conventional (left) and inverted (right) all-PSCs. Reproduced from ref. 137 with permission from Wiley-VCH, copyright 2016. (b) The XRD pattern of the annealed **P155** film on the glass substrate (left) and the ordered lamellar packing state deduced from the diffraction patterns (right). Reproduced from ref. 144 with permission from the American Chemical Society, copyright 2013. (c) *J*–*V* characteristics (up) and EQE spectra (bottom) of the **P161**- and **P162**-based solar cell devices, with the IT-4F as the acceptor material. Reproduced from ref. 148 with permission from Wiley-VCH, copyright 2019. (d) The output (up) and transfer (bottom) characteristics of **P161** and **P162**. Reproduced from ref. 148 with permission from Wiley-VCH, copyright 2019. (e) 2D-GIXRD patterns of the neat **P167** film (up) and **P167**:PC_71_BM blend film (bottom). Reproduced from ref. 153 with permission from the Royal Society of Chemistry, copyright 2015.

As is well known, the extended and electron-deficient acceptor unit of D–A type polymers can adjust the FMO energy levels and bandgaps, which are beneficial for their device performances. Thus, with the π-extended dithienocoronenediimide as the acceptor, **P148** and **P149** were synthesized to achieve a high degree of regioregularity.^139^ Because of the poor solubility of **P149**, the additional alkylation of the donor was carried out in polymer **P150**, which gave it more widened and red-shifted absorption bands compared with those of **P148**. The electron-rich nature of thiophene fused in dithienocoronenediimide led to the higher HOMO/LUMO energy levels of **P148** and **P150** than those of **P144-P147**. The planar molecular backbone, enhanced regioregularity of dithienocoronenediimide, and the narrow energy gaps eventually provided **P148** and **P150** with ambipolar transport behaviors with higher electron mobilities than those of their PDI-based counter polymers. To avoid the high-rigidity-caused poor solubility of polymer **P149** and to modulate the miscibility with other polymers for high-performance OSCs, Yang and co-workers reported a polymer **P151**, comprising asymmetric thianaphthene-fused perylenediimide (TDI).^140^ The slightly relieved backbone rigidity provided the polymer with good solubility, thus forming uniform blend film morphology with a smooth surface, facilitating a higher PCE of 4.70% than its PDI- or dithienocoronenediimide-based reference polymers did. It should be pointed out that the advantages that the extended planar backbone could bring to the polymers were much more important than the possibly resulting poor solubility, which was easier to modify, for instance, **P152** and **P153**, which were based on naphthodiperylenetetraimide (NDP) units with two fused PDI units.^141^ The super rigid and *π*-extended backbone led to improved backbone coplanarity and the delocalization of π-electrons, with their LUMO energy levels below −4.10 eV. Both the polymers were blended with PTB7-Th and behaved well in their OSC devices with the highest PCE of 6.39%. Such preferable performance was surpassed by another NDP-based polymer, **P154**, in which NDP monomers were connected with vinylene units.^142^ The larger polycyclic NDP units provided the corresponding polymers with tunable chain flexibility and an improved polymeric packing state. The AFM images revealed a smooth and uniform blend film of **P154** with small root-mean-square (RMS), leading to better contact between the active layer and the interfacial electrode. The PTB7-Th:**P154** blend film afforded a high PCE up to 8.59%, proving the efficiency of the fusion strategy to improve the PCE values. Thereafter, more fused derivatives were reported. Recently, Yu and co-workers developed several novel small molecules, in which two NDP units were fused with the benzene rings of IID, BDOPV, *etc.*^143^ All the extended building blocks had narrower band gaps than those of their respective fused inserting units, implying their potential usage in the polymer backbone. In addition to extending the NDI or PDI unit cores laterally, the longitudinally extended tetraazabenzodifluoranthene diimide (BFI) monomers in **P155** and **P156** could also evenly distribute the FMO but in the perpendicular direction to the mainchain.^144^ The vertical extension also provided the two polymers with wide and NIR absorption regions just as the lateral extension could achieve. Moreover, both polymers had orderly packed films, especially for the **P155** film, which had ordered lamellar crystalline with the *d*-spacing (2.6 nm) roughly the same as the backbone length of the BFI unit (Fig. 14b). The n-type transport properties were achieved for **P155** and **P156** with the highest mobility of 0.30 cm^2^ V^−1^ s^−1^ being achieved by **P155** due to its good solubility and high quality of the thin film.

The above NDI, PDI, and their electron-withdrawing derivatives were always used in the polymer main conjugated backbones to build up the connection of donor and acceptor units. Creatively, Drewniak and co-workers introduced NDI and PDI units in the sidechain of **P157** and **P158**, respectively.^145^ Such attempts provided the two polymers with a moderate PCE of around 0.03% in their OSC devices (Table 1). However, introducing the donor unit at the farther position of the main chain backbone could potentially form the interaction between donors and acceptors from different polymer chains during the chain packing process and might be a feasible method to enhance the interchain charge transport in the near future.

As summarized above, a large number of polymers with favorable performances in OFETs and OSCs were reported, whereas there are still some problems that existed. Limited by the molecular structure, there are too many byproducts that appear during the synthesis of the brominated PDI monomer, which directly reduces the yield of the desired product and makes it hard to be purified. Besides, PDI and its derivatives have the same problem in steric hindrance as the NDI does when connected with other units. The resulting poor molecular backbone planarity ultimately led to moderate or even low device performances for those polymers.

### 3.3. Polymers based on other imide-functionalized units

In addition to the widely investigated NDI and PDI units, there are also some other imide-functionalized electron-deficient building blocks, such as phthalimide (PHI), thieno[3,4-*c*]pyrrole-4,6-dione (TPD), and bithiophene imide (BTI) units.

Different from NDI and PDI units, the PHI unit is less electron-deficient and showed absorption in the short wavelength range, thus having the potential to obtain polymers with wide band gaps and achieve unipolar FET performance. In 2009, the PHI unit was firstly introduced into polymers for optoelectronic devices by Guo and co-workers and the corresponding polymer eventually afforded the highest *μ*_h_ of 0.28 cm^2^ V^−1^ s^−1^.^146^ Then the fluorinated PHI unit was utilized to synthesize **P159**, which had lower FMO energy levels than those of PHI-based reference polymer **P160**.^147^ Both the polymers exhibited good OSC performances with the highest PCE value of 8.31% for **P160** and 9.48% for **P159**, with their *V*_oc_ over 0.90 V (Table 1). Thereafter, fluorinated benzothiadiazole and alkylated thiophene units were introduced into the backbones of **P161** and **P162**.^148^ In comparison with **P159** and **P160**, the acceptor units in polymers **P161** and **P162** greatly lowered their HOMO/LUMO energy levels and achieved narrower band gaps. Moreover, the alkylated thiophene units also facilitated a better blend film miscibility with a highly interpenetrating and continuous network. Because of these advantages, both **P161** and **P162** showed favorable PCE values of 13.31% and 12.74%, respectively (Fig. 14c). Apart from the high PCE, two polymers afforded unipolar p-type transport performances with the hole mobilities of 0.63 and 0.93 cm^2^ V^−1^ s^−1^ for **P161** and **P162**, respectively (Fig. 14d), due to their better crystallinity and delocalized π-conjugation. These results revealed the effectiveness of fluorine introduction and the dual acceptor strategy in improving the OSC performances.

A new member of the family of PHI-derived monomers, 4,8-di(thien-2-yl)-6-octyl-2-octyl-5*H*-pyrrolo[3,4-*f*]benzotriazole-5,7(6*H*)-dione (TZBI), for constructing high-performance polymers was reported by Lan and co-workers.^149^**P163** featured a wide optical band gap of 1.81 eV and high HOMO/LUMO energy levels. After thermal annealing and the application of the water/alcohol interlayer in the OSC devices, **P163**:PC_71_BM gave a high PCE of 8.63%, which was surpassed by two of its analogs **P164** and **P165**.^150^ Both the polymers showed apparently lowered FMO energy levels and almost the same optical band gaps as **P163**. Because of the smooth film surface of their blend films and relative balanced hole/electron mobilities, **P164** and **P165** afforded the max PCE values of 10.24% and 12.12%, respectively. These results also demonstrated that the branched side chain strategy and fluorination together could improve the performance of OSCs by facilitating ordered film morphology and more balanced transport performance. Similar to the PHI unit, thieno[3,4-*c*]pyrrole-4,6-dione (TPD) was structurally simple, symmetric, and planar, which was preferable for semiconducting polymers. The first TPD-based polymer, **P166**, was firstly synthesized in 2009 and afforded a PCE of 5.5%.^151^ Thereafter, the **P166**-based ternary blend film with PBDTTT-CT and PC_71_BM exhibited a much-enhanced PCE of 9.3%, attributed to the good miscibility and favorable morphology of the ternary blend film.^152^ When the thienothiophene-flanked TPD and thiophene units are combined, the polymer **P167** was obtained.^153^ In comparison with the neat **P167** film, the **P167**:PC_71_BM blend film, having an additional 1,8-diiodooctane (DIO), provided an enhanced crystallinity, a smaller *π*–π stacking distance of 3.69 Å, and more percentage of the face-on orientation component (Fig. 14e). The appearance of more face-on stacking was preferable for charge carrier transport, thereby achieving a higher PCE up of 9.21%. To achieve a larger planar backbone and stronger electron-withdrawing ability, the bithieno[3,4-*c*]pyrrole-4,6-dione (BiTPD) unit, with two adjacent TPD units, was incorporated into polymers **P168**, **P169**,^154^ and **P170**.^155^ The sulfur–oxygen interaction in BiTPD supported three polymers with good planarities. **P168**- and **P169**-based TGBC FET devices exhibited p-type transport performances with their highest hole mobilities of 0.74 and 0.32 cm^2^ V^−1^ s^−1^, respectively. The higher mobility of **P168** could be associated with the evenly dispersed granular grains in its annealed film and higher crystallinity. Moreover, benefiting from the face-on packed blend film and the induced polymer vertical π-overlap, the **P170**-based OSC afforded the highest PCE of 14.2%, which is much higher than those of **P166** and **P167**. Similar to the BiTPD structure, which directly combined two TPD units, the TPD and NDI units in **P171** and **P172** were also connected *via* the thiazole unit in different ways.^156^ Though **P171** and **P172** had comparable FMO energy levels, **P172** gave a more torsional backbone (Fig. 16a). Interestingly, both polymers afforded unipolar n-type transport performances whereas in different orders of magnitude. The backbone-distorted **P172** exhibited a much higher electron mobility of 2.55 cm^2^ V^−1^ s^−1^ than the 0.06 cm^2^ V^−1^ s^−1^ of **P171**. Such a disruptive phenomenon could be attributed to the more ordered film packing state and higher film crystallinity with multiple Bragg peaks of the **P172** film (Fig. 16b). These results were opposite to the general cognition of the relationship between the polymer backbone planarity and the FET performance. Considering their different film morphologies and electron mobilities, the most key factor in achieving high electron mobilities could be the good film morphology formed during the process of fabricating the device rather than the planar backbone.

The imide groups of the PHI and TPD units were both constructed in a five-membered ring, which had limited π-extension and modifiable position. Therefore, the *N*-alkyl-2,2′-bithiophene3,3′-dicarboximide (BTI) unit built up its imide structure in a seven-membered ring with two fused thiophene units. Since the first report in 2008,^157^ the BTI unit has been increasingly investigated and had derived many types of monomers, among which dithienylthienothiophenebisimide (TBI) was the most common one to see. With two fused BTI units, TBI was equipped with two imide groups and the more extended π-conjugation. A series of polymers **P173-P176** were prepared based on TBI by Saito and co-workers.^158^ All the polymers exhibited good FET performances but different charge polarities. **P173** and **P174** showed p-channel transport behaviors while ambipolar and n-type transport performances were achieved by **P175** and **P176**, respectively. Moreover, the face-on orientation in the **P174**/PC_71_BM blend film was beneficial for its hole transport and charge collection, thus facilitating the highest PCE of 8.0% among those polymers. More recently, Shi and co-workers developed two all-acceptor containing polymers **P177** and **P178** with considerable performances in both OFETs and OSCs.^159^ The homo-polymer **P177** and polymer **P178** showed apparently lowered LUMO energy levels relative to **P173-P176**, which provided them with unipolar n-type transport performances with the respective max electron mobilities of 3.10 and 1.23 cm^2^ V^−1^ s^−1^, respectively (Fig. 16c). Moreover, compared with their neat films, the apparent face-on orientations with stronger (010) scattering peaks were observed in both blend films of **P177** and **P178**. As the electron-accepting materials in the OSC devices, **P177** and **P178** exhibited a PCE of 6.67% and 8.61%, respectively (Fig. 16d). Compared with the polymer derived from the condensation of BTI and hexamethylditin units, **P177** obtained from BTI and distannylated BTI units had a higher *M*_n_ of about 35.5 kDa and showed a smoother film morphology with small RMS roughness, thereby reducing the carrier traps in the polymer film.

Besides the fusion strategy in the TBI monomer, two directly connected BTI units were also used in **P180**.^160^ When compared with the more rigid TBI-based **P179**, **P180** had more blue-shifted absorption spectra, despite their comparable HOMO and LUMO energy levels. Besides, the fused BTI units enabled the **P179** film to strongly aggregate, thereby facilitating continuous domains in its blend film. Such preferable properties led to more efficient exciton dissociation and charge transport. Therefore, **P179**-based FET devices showed a *μ*_e_ of 1.13 cm^2^ V^−1^ s^−1^ and afforded a PCE up to 6.85% in its OSC devices. Both performances were much better than those of polymer **P180**. It was not strange for the unideal properties of **P180** when considering its single bond connected BTI units, which led to a sine-wave shaped backbone rather than the linear shape of **P179** (Fig. 16e). Thereafter, the further fluorine-modified **P181** displayed much improved performances in both OFET and OSC devices compared to those of **P180**.^161^ The incorporation of fluorine atoms formed multiple S⋯F intramolecular interactions thus promising a more planar backbone like the TBI unit in **P179** to afford effective π-conjugation and strong intermolecular packing. Eventually, **P181** displayed considerably enhanced OFET performance with a *μ*_e_ of 2.73 cm^2^ V^−1^ s^−1^ and a high PCE of 6.50% in its OSC device, both of which were greatly enhanced in comparison with **P180** and comparable to those of **P179**. Because of the electron-donating nature of the thiophene rings, the TBI unit actually was not electron-deficient as other imide-containing acceptors, which could also be concluded from the high LUMO energy levels of corresponding polymers. Therefore, the polymer **P182** was prepared based on the modified building block f-FBTI2-Br, which has a fluorine atom substituted at the β-positions of thiophene in the TBI unit.^162^ The LUMO energy level was slightly lowered to −3.46 eV and the fluorinated-TBI unit showed improved backbone planarity compared to the unfluorinated one, with a smaller dihedral angle of 0° between two BTI units (Fig. 16f). In comparison with the preferential edge-on orientation of the **P179** film, the fluorination provided the **P182** film with a face-on dominating bimodal orientation with a distance of 3.6–3.7 Å, thus leading to its further enhanced PCE of 8.1%. As the other most used electron-withdrawing atom, nitrogen was also embedded in the TBI unit of polymer **P183**.^163^ The S⋯N intramolecular interaction greatly optimized the planar conformation of the thiazole-based TBI unit thereby facilitating a dense molecular packing state with a π-stacking distance of 3.35 Å (Fig. 16g). Different from the fluorinated TBI unit in polymer **P182**, the thiazole-based imide in **P183** could not only lower the FMO energy levels but also adjust the dihedral angles between two adjacent BTI units to almost 0° without sacrificing the planarity of the BTI core, thus forming a compact π-stacking distance of 3.65 Å. The off-center spin-coated **P183** film showed the highest electron mobility of 1.61 cm^2^ V^−1^ s^−1^. The high reactivity of the distannylated BTI unit enabled novel acceptor–acceptor polymers, which were favorable for perovskite solar cells (PVSCs) due to the potentially improved electron extraction and simultaneously optimized hole-blocking property compared with the D–A polymers. Therefore, the NDI and PDI units were chosen to replace one of the BTI units in **P177** and finally afforded the polymers **P184** and **P185**, respectively.^164^ Both polymers were applied in perovskite solar cells (PVSCs) as electron transport materials to replace PC_61_BM and exhibited remarkable PCE values of 19.5% and 20.8% for the **P184**- and **P185**-based devices, respectively. Besides, the high hydrophobicity of two polymers protected the perovskite active layer from the penetration of moisture, thereby promising outstanding device stability, especially for **P185**, whose PCE maintained 90% of the initial value after 70 hours.

**Fig. 15:**
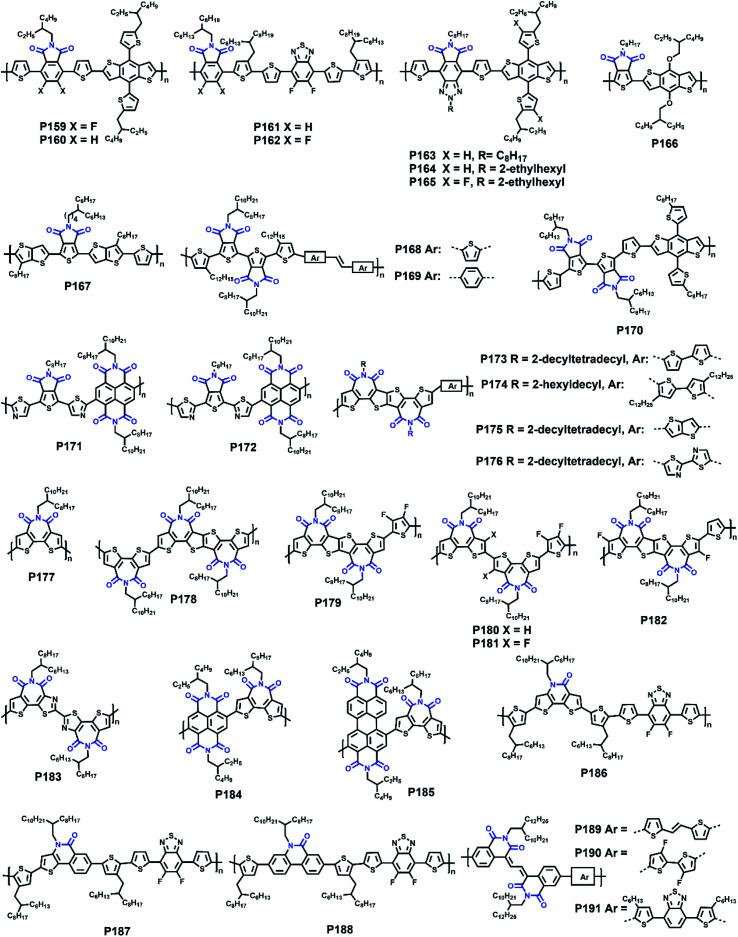
The molecular structures of polymers **P159–P191**.

The BTI unit was mostly developed based on the seven-membered imide ring, as shown in Fig. 15, the polymers **P186-P188** were built up with tricyclic aromatic six-membered lactam monomers.^165^ All their acceptors contained six-membered lactam rings with fused benzene or thiophene moieties. Three polymers showed similar FMO energy levels and band gaps but different performances in OSC devices under the same conditions. Attributed to the less bimolecular recombination and more balanced hole/electron mobilities, **P187** afforded a higher PCE of 10.16% than 8.61% and 8.47% of **P186** and **P188**, respectively.

**Fig. 16:**
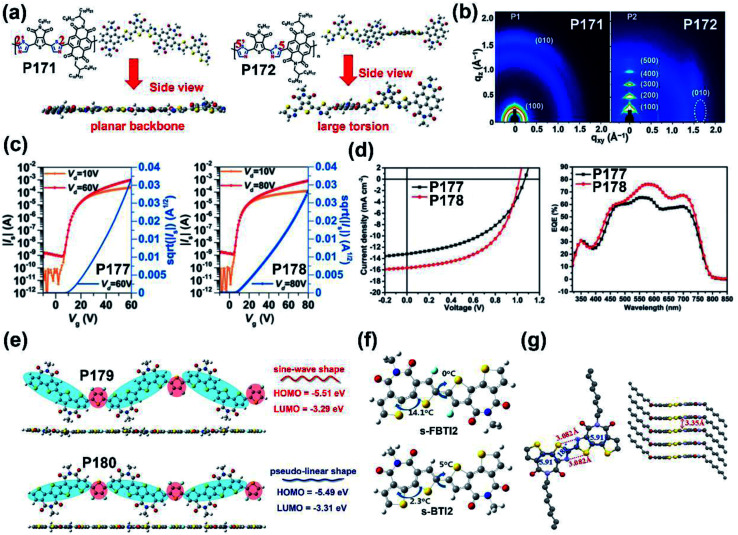
(a) The molecular structures of **P171-P172** and their side views of the optimized backbone geometries. Reproduced from ref. 156 with permission from Wiley, copyright 2019. (b) 2D-GIXRD patterns of **P171-P172**. Reproduced from ref. 156 with permission from Wiley, copyright 2019. (c) The transfer characteristics of **P177**- and **P178**-based OFETs. Reproduced from ref. 159 with permission from Wiley, copyright 2020. (d) *J–V* characteristics and EQE spectra of the PTB7-Th: **P177**(**P178**)-based all-PSCs. Reproduced from ref. 159 with permission from Wiley, copyright 2020. (e) The simulated backbone conformation with the minimum energy and the backbone curvatures of the **P179** and **P180** trimers. Reproduced from ref. 160 with permission from Wiley, copyright 2017. (f) The DFT-calculated structures of the dual BTI monomers before (bottom) and after fluorination (up). Reproduced from ref. 161 with permission from Wiley, copyright 2018. (g) Single crystal structure (left) and the π-stacking distance and packing state (right) of the repeating unit of **P183**. Reproduced from ref. 163 with permission from Wiley, copyright 2018.

Compared with the seven-membered imide, the above three six-membered building blocks had one less carbonyl group, which led to the much higher FMO energy levels of their corresponding polymers in comparison with most BTI-based polymers. Therefore, Huang and co-workers developed a new building block, EBIQ, based on the acceptor unit of **P188**.^166^ The replacement of one fused phenyl ring with the carbonyl group provided the EBIQ with an additional O⋯H hydrogen bond and preferable planarity, promising small π-spacing distances of around 3.50 Å for **P189-P191** (Table 1). With the bimodal orientation for their films, all three polymers showed ambipolar transport behaviors, and the highest hole/electron mobilities of 0.138/0.074 cm^2^ V^−1^ s^−1^ were achieved by **P190** due to its smallest π–π distance of 3.47 Å provided by enhanced dispersion interactions.

## 4. Summary and outlook

Recent two and three decades have witnessed the rapid development of semiconductor polymers for applications in OEFTs and OSCs. Among the widely investigated polymers, a large number of D–A alternating polymers have been designed and synthesized, which could be attributed to the prosperous development of donor and acceptor units, especially easily adjustable electron-deficient building blocks.

In the first part, we discuss lactam-containing polymers based on electron-withdrawing building blocks, containing DPP, IID, and their derivatives. Because of the variety of adjustable flanking units, DPP-based polymers are equipped with numerous optoelectronic properties, such as good solubility offered by furan, deep-lying FMO energy levels enabled by pyridine, thienothiophene-orientated extended backbone conjugation, individually adjustable LUMO energy levels offered by thiazole, selenophene-induced compact polymer chain packing state, *etc.* All the above bridges can be incorporated asymmetrically into the DPP core because of their potentially combined favorable properties. Direct modifications of the DPP core, including the exchange of lactam nitrogen and carbonyl groups, insertion of the benzene/naphthalene ring, the introduction of half-fused lactam, and the conversion from the fusion strategy to the double-bond connection, can still exert their potential in optimizing the specific properties of polymers. Different from DPP-based building blocks, most derivatives and analogs of the IID core are developed with relatively better planarities due to many intramolecular interactions (*e.g.*, S⋯O) and O⋯H hydrogen bonds. By structurally adjusting the indolinone units with substituted electron-withdrawing heteroatoms, the obtained polymers have lowered energy levels and achieve high balanced ambipolar transport performances. The extension of the indolinone unit can efficaciously facilitate effective π-orbital overlap and preferable face-on orientation in the polymer films for exhibiting high PCE of OSC devices. Further modification with a variety of inserting units destroys the former planar backbone in IID and constructs new monomers also featuring planar backbones and additionally more delocalized molecular orbitals, more alkylation sites, and decreased sidechain density for an ordered sidechain self-assembly process.

In the second section, we discuss the recent structural evolution of NDI, PDI, BTI, and other imide-functionalized building blocks, as well as the corresponding polymers. In comparison, NDI and PDI units have more rigid backbones than those of DPP and IID cores. Precisely because of their special structures, the laterally extended conjugation can promise good planarity and relieve the steric effect between NDI/PDI and the adjacent units at the same time. This provides those polymers with improved mainchain stacking and more percentage of face-on orientation in the films, let alone their enhanced OSC performances. For the backbone extension in the vertical direction, it shows effective adjustment in achieving a narrow band gap with the relatively low-lying LUMO energy level, which favors ambipolar charge carrier performances. Moreover, PHI, BTI, and other imide-containing units are also introduced. Whether the bonding or the fusion strategy, *e.g.*, BiTPD and TBI, polymers can be easily constructed to have more imide moieties in their backbones, due to the structural simplicity of those single imide-functionalized units. This provides the polymer with a staggered sidechain arrangement, which is different from the symmetrical alkylating position in NDI/PDI units. Such a combination contributes to ordered layer-to-layer lamellar stacking and interchain packing simultaneously.

To sum up, several methods have been developed to promote the evolution of those polymer backbones: (i) incorporating electron-withdrawing atoms or groups, (ii) extending the polymeric π-conjugation length, (iii) side chain engineering and (iv) choosing the appropriate donor–acceptor pair. All these structural modifications are mainly focused on two aspects, one of which is decreasing the charge carrier injection barrier and the other one is promoting the charge carrier transport in the polymer film.

Despite the numerous kinds of polymers and their enhancing device performances in OFETs and OSCs, there are still several things that need to be settled down in the next few years. First, the dominant packing state of the polymer chain is uncontrollable. The available sidechain and mainchain orientations, which are in line with the expectations, help investigate the relationship between the chemical structure and the intra- or interchain charge transport. Second, the flexible high-performance semiconductor polymers are still lower in number. The flexibility and conductivity of semiconducting polymers get all the attention. Though there are several pieces of research on flexible polymers and corresponding flexible devices that have been reported, OFET or OSC devices based on intrinsically flexible semiconductor polymers can hardly afford comparable performances with their unstretched or unbending counterparts. So far, long side chain substitution has been proved to be able to improve polymer flexibility due to the ability to counteract the strain energy during the deformation process. Such modification neither affects intrachain carrier transport properties nor prevents interchain aggregation and it deserves further study. Finally, the shape of the polymer backbone, which is easily ignored, should also be considered as another key factor affecting the device performance. Many aspects are associated with the shape formation of polymer backbones, such as the shape of the building block, the bonding method and relative position, and the conformational stability. All those adjustable aspects can be fine-tuned for numerous shape-changeable polymers.

In addition to those mentioned above, the mobility hype is another key point worthy of being paid attention to. As is known, an ideal OFET device should contain multiple features, like negligible metal/semiconductor contact resistance, no diffusion, and biasing-independent mobility. Nevertheless, it is hard to fabricate such a perfect FET device due to the existence of Schottky barriers at the metal/semiconductor contact and other commonly existing device disorders. It is widely accepted that most OFET charge carrier mobilities (*μ*) are mainly extracted by the conventional method in linear and saturation regions. Owing to the respective underestimated and overestimated mobilities obtained from linear and saturation regions, such swinging characteristics of the extracted mobility make the analysis of intrinsic charge carrier mobilities difficult.^167^ Therefore, neither of the two mobilities reflects the intrinsic *μ* of semiconductors, especially when a non-ideal electrical behavior existed. It is noted that we should make a reasonable assessment of those seemed “ultra-high” mobilities probably extracted from the “kink” or “double-sloped” transfer curves, whose drain-source current is not changing linearly as the gate voltage (*V*_G_) increasing. Such non-ideal transfer curves are usually caused by the aforementioned contact resistance and always lead to a substantially inaccurate *μ*. According to the relative values of contact resistance (*R*_c_) and channel resistance (*R*_ch_), the extracted *μ* can be different. When *R*_c_ is larger than *R*_ch_, the rapidly decreased *R*_c_ in the low *V*_G_ region leads to a steep increase in the drain-source current, which means that *μ* obtained from the low *V*_G_ with the steeper slope can hardly reflect the intrinsic transfer behavior and is overestimated. In this situation, the more valid *μ* should be extracted from the high *V*_G_ region. On the other hand, when *R*_c_ is smaller than *R*_ch_, the obtained *μ* is always underestimated whether in the low or high *V*_G_ region.^168^ Despite their referential functions, such pitfall of mobility should be avoided for the improved reliability of the mobility. To reduce the impact of *R*_c_ on the valid *μ*, we should pay more attention to the electrode materials and the FMO energy levels of polymer semiconductors. Apart from these two key points, the gate dielectric is another noteworthy factor in the futural optimization of more reliable mobilities. It can affect the polymer film morphology to achieve different charge trapping therefore shielding the gate field, which leads to greatly varied *R*_c_.^169^ In addition to making changes at the experimental level, another extracted *μ* called the low-field mobility extracted by the *Y*-function method appeared to achieve less mobility misinterpretation and higher reliability. It has been pointed out in recent years that the mobilities obtained from the *Y*-function method are closer to the effective mobilities in comparison with those extracted by the conventional method whether in linear or saturation regions.^170^ It will be more delightful to build up strong reliability in the field than chasing the so-called “ultra-high performance” by incorrect methods.

## Author contributions

G. Y. worked out the overall plan of this review article. The manuscript was written through contributions of all authors.

## Conflicts of interest

There are no conflicts to declare.
